# Emergence of Connectivity Motifs in Networks of Model Neurons with Short- and Long-Term Plastic Synapses

**DOI:** 10.1371/journal.pone.0084626

**Published:** 2014-01-15

**Authors:** Eleni Vasilaki, Michele Giugliano

**Affiliations:** 1 Department of Computer Science, University of Sheffield, Sheffield, United Kingdom; 2 Department of Biomedical Sciences, University of Antwerp, Wilrijk, Belgium; 3 Brain Mind Institute, Swiss Federal Institute of Technology of Lausanne, Lausanne, Switzerland; SUNY Downstate MC, United States of America

## Abstract

Recent experimental data from the rodent cerebral cortex and olfactory bulb indicate that specific connectivity motifs are correlated with short-term dynamics of excitatory synaptic transmission. It was observed that neurons with short-term facilitating synapses form predominantly reciprocal pairwise connections, while neurons with short-term depressing synapses form predominantly unidirectional pairwise connections. The cause of these structural differences in excitatory synaptic microcircuits is unknown. We show that these connectivity motifs emerge in networks of model neurons, from the interactions between short-term synaptic dynamics (SD) and long-term spike-timing dependent plasticity (STDP). While the impact of STDP on SD was shown in simultaneous neuronal pair recordings *in vitro*, the mutual interactions between STDP and SD in large networks are still the subject of intense research. Our approach combines an SD phenomenological model with an STDP model that faithfully captures long-term plasticity dependence on both spike times and frequency. As a proof of concept, we first simulate and analyze recurrent networks of spiking neurons with random initial connection efficacies and where synapses are either all short-term facilitating or all depressing. For identical external inputs to the network, and as a direct consequence of internally generated activity, we find that networks with depressing synapses evolve unidirectional connectivity motifs, while networks with facilitating synapses evolve reciprocal connectivity motifs. We then show that the same results hold for heterogeneous networks, including both facilitating and depressing synapses. This does not contradict a recent theory that proposes that motifs are shaped by external inputs, but rather complements it by examining the role of both the external inputs and the internally generated network activity. Our study highlights the conditions under which SD-STDP might explain the correlation between facilitation and reciprocal connectivity motifs, as well as between depression and unidirectional motifs.

## Introduction

Our higher cognitive functions and our memories are believed to be encoded in the wiring diagram of the brain. The technological efforts and the recent initiatives in mapping and understanding the emergence and development of this diagram, the “connectome” [1], undoubtedly represent the cutting edge of research in neuroscience and are confronted with many challenges.

Studies at the microcircuit level revealed that connectivity is non-random [2], [3] and, in particular, specific cellular connectivity *motifs* have been found in percentages well above chance level. Some of these studies have also been able to provide physiological information about the neurons and synapses that are involved in the formation of such motifs [2]–[5]. Besides revealing the molecular identity of neurons, such information includes the properties of activity-dependent short-term [6], [7] or long-term plastic changes in synaptic efficacy [8], [9] as well as the synaptic rewiring [10], [11]. These physiological details are of great significance, as the transmission of information between neurons takes place by means of more than mere “connectors.” For instance, synaptic efficacy undergoes short-term dynamics (SD), quantified as transient and reversible facilitation or depression of postsynaptic responses, upon repeated presynaptic activation [6], [12].

Of interest for our study, short-term facilitation and depression were found to correlate to specific, pairwise, connectivity motifs: neurons connected by synapses exhibiting short-term facilitation form predominantly reciprocal motifs; neurons connected by synapses exhibiting short-term depression form unidirectional motifs. This correlation was observed in glutamatergic microcircuits of rodent cortex [4] and the olfactory bulb [13], but the mechanisms responsible are largely unknown.

Inspired by a theory on the relationship between neural code and cortical connectivity [14], we hypothesize that interactions between short-term and long-term synaptic plasticity might contribute to the emergence of pairwise connectivity motifs observed in the experiments. We explore this hypothesis *in silico* by combining together existing phenomenological models describing spike-timing dependent long-term synaptic plasticity (STDP) [15] and SD [16], as well as by using analytical arguments, well-established in the literature [14], [15], [17]–[20].

We find that, by SD-STDP interplay alone, identical networks of Integrate-and-Fire model neurons [21] evolve reciprocal motifs if their synapses are facilitating and unidirectional motifs if the synapses are depressing. The key ingredients to explain the evolving motifs are the facts that (i) networks with facilitating synapses fire at higher frequencies than networks with depressing synapses, and (ii) the model of STDP captures both a correlational “pre-post” *temporal* mode at low firing rates and a “Hebbian” *rate* mode at high firing rates [22].

The hypothesized SD-STDP interplay for connectivity motif correlation emergence might be one of the possible mechanisms that contribute to shape brain microcircuitry, complementing an existing theory [14] by focusing on the role of internally generated network activity, in addition to external inputs.

## Results

We demonstrate in simulations and by analytical arguments that two homogenous networks of model neurons, identical in every other aspect but the type of connecting synapses, i.e., facilitating or depressing, will evolve to two distinct connectivity profiles, under identical external stimulation: the facilitating network will develop reciprocal motifs while the depressing network will develop unidirectional motifs.

The key mechanisms in this finding are (i) the SD, which results in networks with facilitating synapses to fire at higher rates than networks with depressing synapses, and (ii) the long-lasting potentiation components (LTP) of the STDP, which above a *critical* firing frequency threshold prevail over the depression components (LTD), regardless of temporal correlations as observed experimentally by Sjoestrom *et al.* (2001).

As in [14] and [2], we focus on stereotypical motifs of strong synaptic efficacies among weak links between recurrently connected neurons, and study how the values of synaptic coupling become large enough that the internal dynamics of the network dominates over external inputs. For instance, the moderately high frequency internal activity of the facilitating networks overrides the external inputs and leads to reciprocal motifs, according to the classic Hebbian associative plasticity. This doesn't happen with the depressing networks, which naturally fire at lower frequency and where external inputs prevail, evolving only unidirectional motifs.

In cases where the external inputs anyway drive neurons to fire strongly, the networks will evolve reciprocal motifs, regardless of the nature of the synapses (see also [14]). This fact strengthens our results, as experimentally one always observes a minor percentage of reciprocal connectivity and depressing synapses, as well as unidirectional connectivity and facilitating synapses [13].

We further extend our results to heterogeneous networks, where both facilitating and depressing synapses are present. We study the conditions under which our results hold, we describe that spatial structure in the initial synaptic efficacies is required, and we propose the conditions by which this structure may be formed.

A subset of these results, limited to toy, homogenous networks under the presence of background noise and extreme initial connectivity (fully reciprocal or fully unidirectional), was earlier reported in [23]. Here we considerably extend and generalise these results, providing a full analysis of the underlying mechanisms.

### Homogenous microcircuits

In the following section we study and analyze small and large scale homogenous networks, i.e., networks where all synapses between neurons are all facilitating or all depressing, and describe the mechanisms underlining the motif formation.

#### A toy, recurrent microcircuit model with weak background external inputs

**Figure 1:**
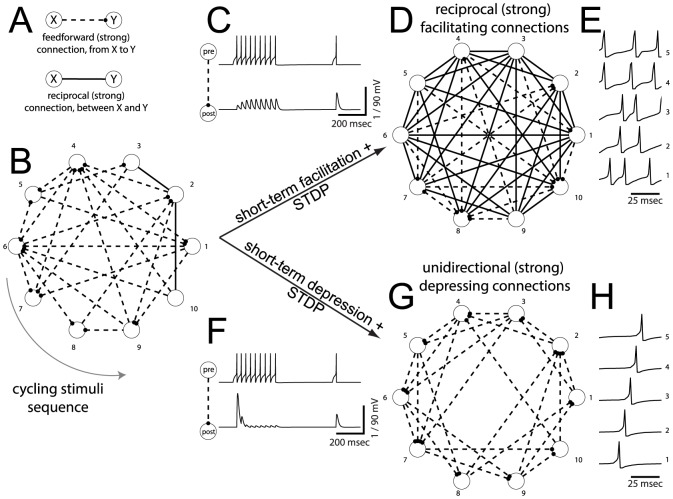
Emergence of connectivity motifs in a toy model network. Unidirectional (reciprocal) strong excitatory connections are indicated (**A**) as dashed (continuous) line segments, representing the topology of the network (**B**). Each model neuron receives periodic spatially alternating depolarizing current pulses, strong enough to make it fire a single action potential. Synapses among connected neurons display (**C**) short-term facilitation of postsynaptic potential amplitudes. Spike-Timing Dependent Plasticity (STDP) leads to strengthened connections and results in a largely reciprocal topology (**D**). Modifying the short-term plasticity profile into depressing (**F**) leads to a largely unidirectional topology shaped by STDP(**G**). Distinct motifs of strong connections arise from short- and long-term plasticities, due to distinct firing patterns (compare **E** and **H**), under identical external stimulation and initial connections. Parameters: 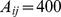 pA, and Methods.

**Figure 2:**
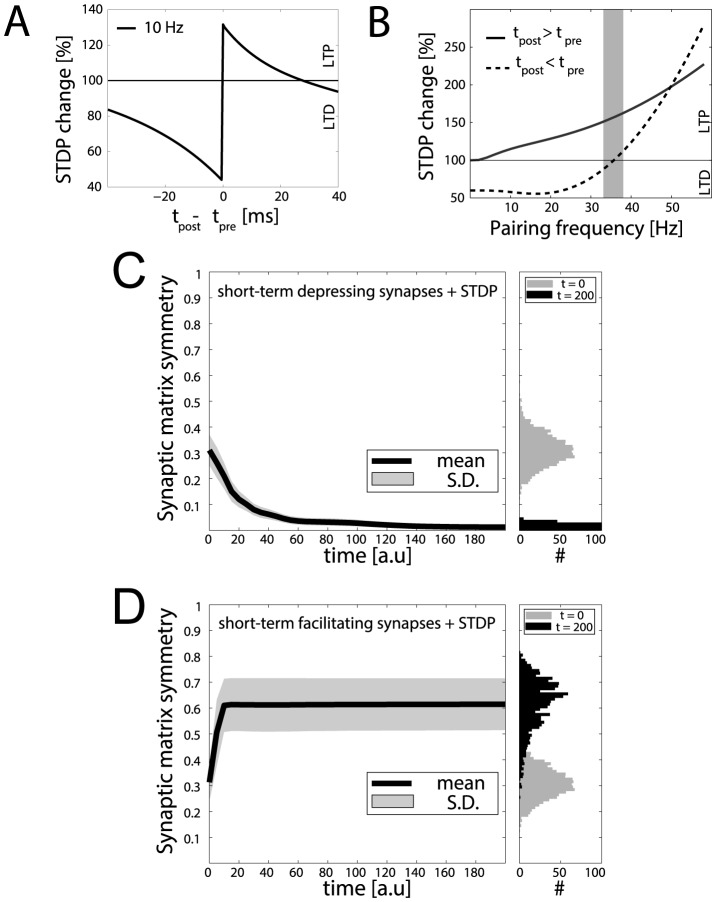
Statistics of motif emergence in a toy model network. When decoupled from recurrent interactions, an isolated model synapse undergoes long-term changes depending on pre- and postsynaptic spike timing (**A**) and pairing frequency (**B**). Above a *critical* frequency (grey shading), spike timing no longer matters and long-term potentiation of synaptic efficacy (LTP) prevails on long-term depression (LTD). Panels **C, D**: The simulations of Fig. 1 were repeated 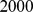 times, each time starting from a random initial topology. STDP progressively induced a persisting non-random reconfiguration of strong connections, quantified across time by a symmetry index (see Methods). Neurons connected only by short-term depressing synapses evolved strong unidirectional connections, corresponding to a low symmetry index. This is displayed in panel **C**, as an average across the 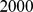 simulations (left panel). Initial and final distributions of symmetry index values are also shown (right panel, grey and black histogram respectively). Neurons connected only by short-term facilitating synapses evolved instead strong bidirectional connections with high symmetry indexes (**D**).

We first consider a simplified representation of an excitatory microcircuit: a network of adaptive exponential Integrate-and-Fire (IF) units. Neurons are connected to each other through excitatory synapses (Fig. 1 B), whose efficacy undergoes short- and long-term plasticity, according to widely studied phenomenological descriptions of short-term synaptic dynamics (SD) [6] and of long-term spike-timing dependent plasticity (STDP) [15] (see Methods). At low firing frequencies, this STDP model reproduces the common temporal correlation window, i.e., the long-lasting plastic change depends on whether the presynaptic neuron fired before the postsynaptic or not (Fig. 2 A). At moderately high firing frequencies, however, the same model captures Hebbian associative plasticity, in the sense that neurons that fire together wire together regardless of their firing timing (Fig. 2 B). This is in agreement with the experiments [22], where above 30–40 Hz LTP prevails on LTD, even when spike-timing *per se* would promote LTD, i.e., 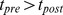. Below that *critical* frequency, LTP or LTD instead reflects causal or anti-causal relationships between pre- and postsynaptic firing times, respectively [9]. Details on implementation and parameters used are described in Methods and in Table 1.

**Table 1:** Parameters employed in the simulations: STDP parameters are as in the *minimal all-to-all* triplet model described in Pfister and Gerstner (2006); short-term depression and facilitation parameters as in (Wang *et al.*, 2006); neuron parameters are as in (Clopath *et al.*, 2010).

Symbol	Description	Value
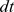	Forward Euler method integration time step	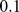 msec
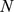	Number of simulated neurons	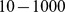
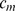	Membrane capacitance	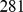 pF
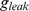	Membrane leak conductance	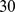 nS
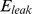	Resting membrane potential	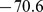 mV
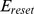	After-spike reset potential	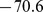 mV
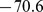	Spike steepness of the exponential IF model	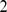 mV
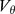	Spike emission threshold of the exponential IF model	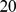 mV
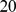	Threshold voltage parameter of the exponential IF model	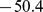 mV
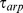	Absolute refractory period	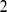 msec
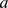	Voltage dependence coefficient of the spike-frequency adaptation	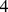 nS
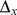	Spike-timing dependence parameter of the spike-frequency adaptation	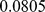 nA
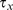	Time constant of the spike-frequency adaptation	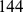 msec
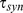	Excitatory postsynaptic currents decay time constant	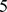 msec
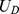	Release probability, for depressing synapses	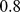
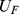	Release probability, for facilitatory synapses	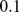
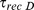	Time constant of recovery from depression, for *depressing* synapses	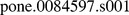 msec
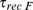	Time constant of recovery from depression, for *facilitating* synapses	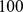 msec
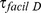	Time constant of recovery from facilitation, for *depressing* synapses	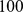 msec
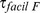	Time constant of recovery from facilitation, for *facilitating* synapses	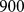 msec
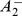	STDP model LTD amplitude for post-pre event	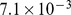
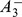	STDP model LTD amplitude for post-pre event (triplet-term)	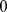
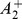	STDP model LTP amplitude for pre-post event	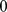
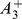	STDP model LTP amplitude for pre-post event (triplet-term)	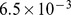
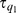	STDP model decay time of presynaptic indicator 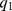	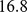 msec
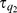	STDP model decay time of presynaptic indicator 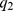	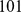 msec
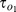	STDP model decay time of postsynaptic indicator 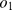	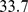 msec
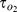	STDP model decay time of postsynaptic indicator 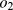	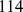 msec
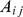	Maximal synaptic efficacy	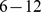 pA
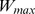	Upper boundary for STDP dimensionless scaling factor 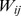	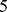
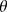	Threshold of the frequency-current response curve for mean-field models	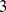
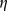	STDP plasticity rate	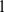

In addition to internally generated synaptic inputs, neurons receive weak external inputs deterministically played back over and over, as a traveling wave of activity (Fig. 1 B). Such a background stimulation imposes spike-timing correlations (as in the *temporal code* of Clopath *et al.* (2010)) and should be regarded an oversimplified generic e.g., thalamic, input with temporally-correlated structure.

We define two microcircuits, identical for all aspects of neuronal properties, maximal synaptic efficacy, anatomical connectivity, and external inputs, with the exception of the SD properties of the synapses. Specifically, one microcircuit includes only short-term facilitating synapses (Fig. 1 C), while the other includes only short-term depressing synapses (Fig. 1 F). Synaptic maximal efficacies, which are initialized as uniformly distributed random numbers, slowly evolve during the simulation into largely non-random configurations of strong links among weaker connections (Figs. 1 D, G), via STDP. At the steady state, these configurations match an experimentally observed co-occurrence: reciprocal motifs emerge in cell pairs more often than unidirectional motifs, when synapses are short-term facilitating; the opposite occurs when synapses are depressing.

**Figure 3:**
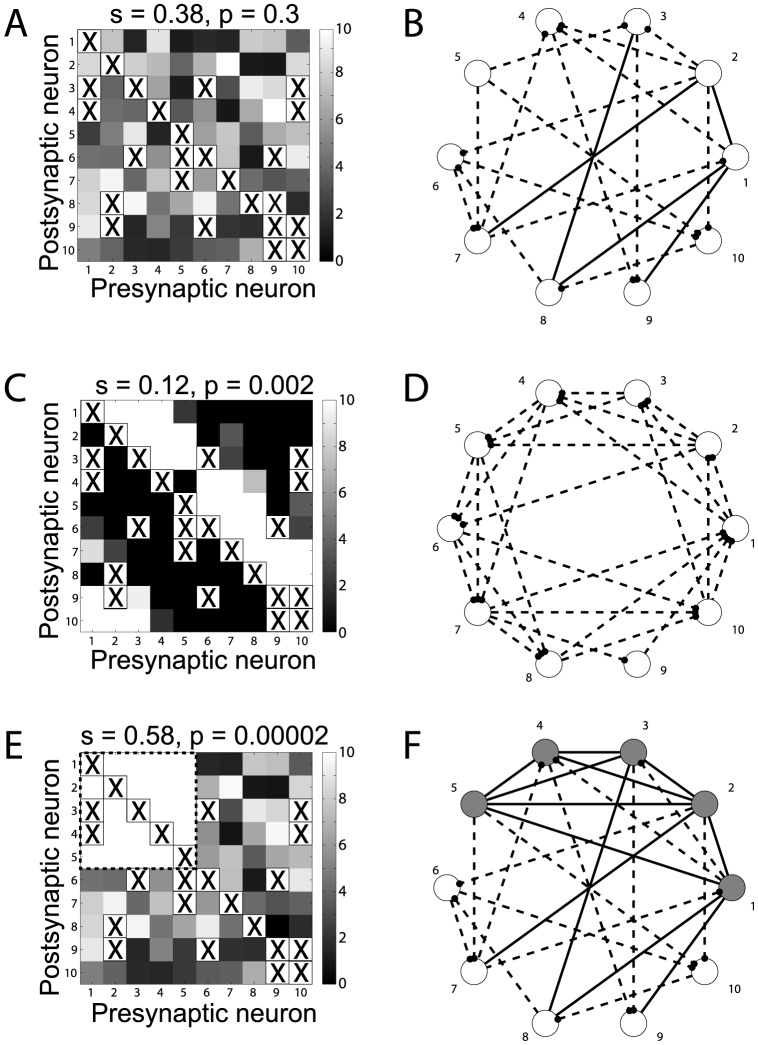
Strong external inputs may drive neurons to high firing rate and induce reciprocal motif emergence even in depressing networks. As in Fig. 2, the synaptic connectivity matrix of a network of ten neurons was randomly initialized and pruned (**A, B**, i.e., pruning is indicated by the “X” symbols). An external weak input, in addition to internally generated activity, contributes to the emergence of non-random unidirectional motifs, resulting in an asymmetric matrix W (**C, D**). However, if five units of the same network (grey circles) are externally stimulated above the STDP *critical* frequency, a non-random connectivity emerges, featuring reciprocal motifs and a symmetric connectivity submatrix (**E, F**; upper left corner, dashed rectangle). The values indicated above panels **A, C, E** represent the symmetry index and its significance.

In our simulations, this is revealed both by direct inspection of the synaptic efficacy matrix 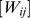 (e.g., see Fig. 3) and by quantification via a connection symmetry index 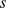 (see Methods). When 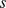 takes values close to 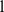, almost all of the existing strong pairwise connections are reciprocal. On the other hand, for values of 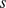 close to 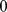, unidirectional or very weak connection motifs prevail.

These results, summarized in Fig. 1 for a sample microcircuit composed by ten neurons, have been confirmed over 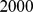 repeated simulations and analyzed in Fig. 2 . Over a slow timescale, which is controlled by the plasticity rate parameter of the STDP model, the interaction of SD and STDP leads to a very small degree of symmetry (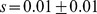, mean 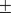 stdev), with high significance (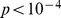) for networks with depressing synaptic connections (Fig. 2 C). Under identical external inputs, SD and STDP lead to a large degree of symmetry (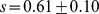, mean 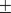 stdev), with, again, high significance (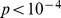) in about 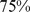 of the networks involving facilitating synaptic connections (Fig. 2 D). In the remaining 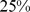 of the cases, the resulting symmetry value was in the range 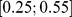, suggesting a degree of variability as found in experiments [4], [13], where the discussed motif correlations are not observed 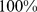 of the time. In this simplified example, the variability is attributed to the sparse structural connectivity imposed *a priori* to result in an irregular firing regime (see Methods). Doubling the simulation time (not shown) led to a very minor reduction of the variability on 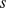, by less than 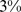 and only for the facilitating synaptic connections, suggesting that stationarity had been already reached. The synaptic connectivity and firing activity configurations of our networks are stable by virtue of the strict boundaries imposed on synaptic efficacy values, as in most numerical implementations of STDP [24]–[26].

It is worthy to note that the microcircuits including short-term depressing synapses collectively fired at 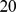 Hz (Fig. 1 H), reflecting the externally imposed firing activity, while the microcircuits employing short-term facilitating synapses fired at 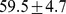 Hz (Fig. 1 E) and exhibited irregular firing patterns, which emerged by the recurrent network structure. We underline that the firing rate of the facilitating network was above the *critical* frequency that separates the “temporal” mode of STDP from its “Hebbian” mode (i.e., as LTD reverts to LTP, see Fig. 2 B). Therefore, for facilitating networks, LTP prevails and the temporal correlations of spike times are no longer important. Similarly, the depressing network fires below the *critical* frequency, and LTP or LTD are determined by the timing of the pre- and postsynaptic spikes. In the following sections, we discuss in detail the mechanism underlining motif formation.

#### Mean-field analysis of homogenous microcircuits

To understand our simulation results, and in particular the difference of internally-generated activity in the two cases, we employ standard Wilson–Cowan firing rate description of neuronal population dynamics (see Eq. 12 [27]). This kind of analysis, whose full details are provided in Text S1, is by no means novel but to the best of our knowledge never reported before in the context of motif emergence.

**Figure 4:**
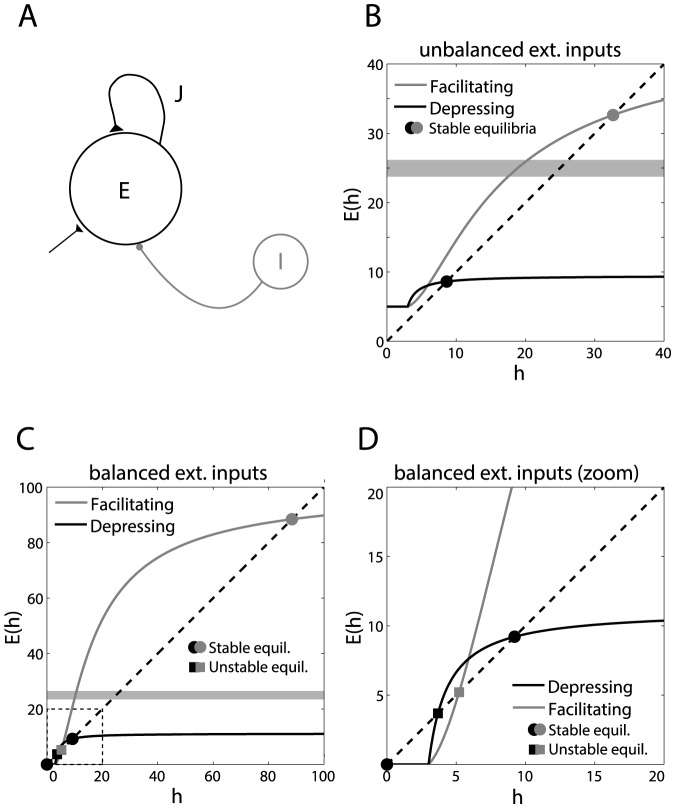
Mean-field analysis of firing rate equilibria, in homogeneous networks without long-term plasticity. The firing rate of homogeneous recurrent networks (**A**), including short-term facilitating synapses or depressing excitatory synapses, was studied by standard mean-field analysis. Average synaptic efficacies are indicated by 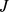. Excitatory and inhibitory external inputs are modeled by a single term, taking positive, zero or negative values. A zero value corresponds to *balanced* excitatory/inhibitory inputs, while a non-zero value corresponds to *unbalanced* excitatory/inhibitory inputs. The steady-state firing rate (i.e., 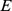, in a.u.) are the roots of the equation 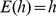 (see Text S1), whose graphical solution is provided (**B–D**), for facilitating (grey) or depressing (black) synapses, without long-term plasticity. Panel **D** is a zoomed view of **C**. (Un)stable firing rate equilibria are indicated by filled circles (squares). Networks with facilitating synapses fire at higher rates than networks with depressing synapses, as emphasized by the grey shading.

We initially consider recurrent networks of excitatory neurons, connected by synapses whose average efficacy 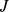 is not a constant but changes on the short term as a function of the presynaptic firing rate, facilitating or depressing (see Fig. 4 A and Eq. 13). For the sake of simplicity, we mimic the effect of feedforward inhibition by an average extra input to the population: we study the case in which this input is zero, i.e., referred to as *balanced* inputs (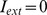 in Eq. 12), or in which it is set to a positive value, i.e., referred to as *unbalanced* inputs, (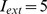 in Eq. 12). Including recurrent inhibition does not qualitatively alter the validity of our conclusions (see Text S1, section 1.4).

The firing rate of the population can be then studied via standard methods of dynamical system theory [28], i.e., analyzing the system of mean-field equations for SD and for neuronal dynamics, and evaluating the stability of their steady-state solutions.

While for simplicity we did not choose the parameters of the rate models to exactly match the IF simulations [29], [30], we are nevertheless able to conclude that homogeneous neuronal populations with short-term facilitating synapses generally fire at higher rates than populations with depressing synapses, for the same values of maximum synaptic coupling and external inputs 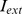, and when engaged in internally-generated reverberating activity [29]. In particular, firing rates are limited by an upper bound that inversely depends on the time constant for recovery from short-term depression (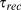 – see Text S1, Eq. S23):
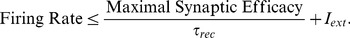
(1)


It follows that, with other parameters being equal, a network with small values of 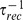, i.e., where the time scale of recovery from depression is very long, would fire slower than a network with comparatively larger values of 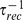, i.e., where the time scale of recovery from depression is very fast or negligible. These two cases correspond to the numerical parameters employed for the networks with depressing and facilitating synapses, respectively (see Table 1 and [4], [13]). For a detailed analysis see section 1.3 of Text S1.

Panels B and C in Fig. 4 illustrate graphically in the state plane *output firing rate* versus *average neuronal input* (i.e., 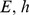), i.e., the actual location of the population equilibria for populations with facilitating or depressing synapses. The stability of each equilibrium point is indicated by different marker symbols: circles for stable, squares for unstable equilibrium points. Similarly to Fig. 2 B, the approximate location of the STDP *critical* frequency has been indicated by a grey shading.

#### A simple mechanism for the emergence of motifs

We have now reviewed that networks with short-term facilitating synapses fire on the average at higher frequencies that depressing networks, for the same external inputs and maximal synaptic efficacies. According to the STDP model implemented here, with parameters as in Table 1, the long-term average change of the maximal synaptic efficacy can be written in a concise form (see [15] and Eq. 16):
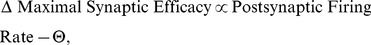
(2)under the assumption that presynaptic and postsynaptic spike trains have Poisson statistics, as hypothesized in the previous paragraph. In Eq. 2, 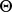 is the *critical* firing frequency threshold of the postsynaptic neurons, above which LTP occurs and below which LTD takes place. If 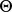 is between the stable equilibria for the population firing rate of the network with depressing synapses and those of the network with facilitating synapses (as in our setup), it follows that the change of maximal synaptic efficacy in the “facilitating” network will be positive while in the “depressing” network will be negative. The maximal synaptic efficacies in the facilitating network will then continuously increase until reaching their upper bound, leading to bidirectional connectivity motifs. On the contrary, the maximal synaptic efficacies in the depressing network will decrease, leading to connectivity motifs that would depend on the spike-timing information only, e.g., as those imposed by the (weak) external background stimulus. At this point, the correlational “pre-post” *temporal* mode of the STDP model comes into play, enforcing unidirectional connectivity motifs to the “depressing” network. Indeed, causality in the spike-timing unavoidably leads to unidirectional reinforcing of either one of connection on a synaptic pair, but never on both simultaneously [14].

In Text S1, we show analytically that under the external stimulus STDP promotes unidirectional connectivity that reflects the asymmetric temporal structure of the inputs, following the analysis of Clopath *et al.* (2010).

#### Positive and negative controls

Our analysis indicates that the motif formation is determined by the firing frequency of the neurons and by the features of the long-term plasticity, i.e., sensitive to both the timing and frequency of spiking activity. Here, we demonstrate these two points (i) by imposing a strong background external input, as in the rate-coding of [14], and (ii) by *ad hoc* altering the physiological dependence of the STDP model on spike timing or on frequency.

We first consider a depressing network with anatomical connectivity and synaptic efficacies randomly initialized as in Fig. 1 (see the Methods section). Figures 3 A, B shows the initial network structure and connectivity. After the exposure to a weak background external input, as in the temporal coding of [14] (i.e., as in Fig. 1), the network evolves only unidirectional motifs, resulting in an asymmetric synaptic efficacy matrix 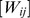, see Figs. 3 C, D. However, if five units of the same network (grey circles) are externally stimulated so that they fire above the STDP *critical* frequency 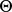, a non-random connectivity emerges, featuring both unidirectional and reciprocal motifs and a symmetric connectivity submatrix (Figs. 3 E, F; upper left corner, dashed rectangle). This demonstrates that external activity can also impose connectivity motifs to the network, as in [14]).

**Figure 5:**
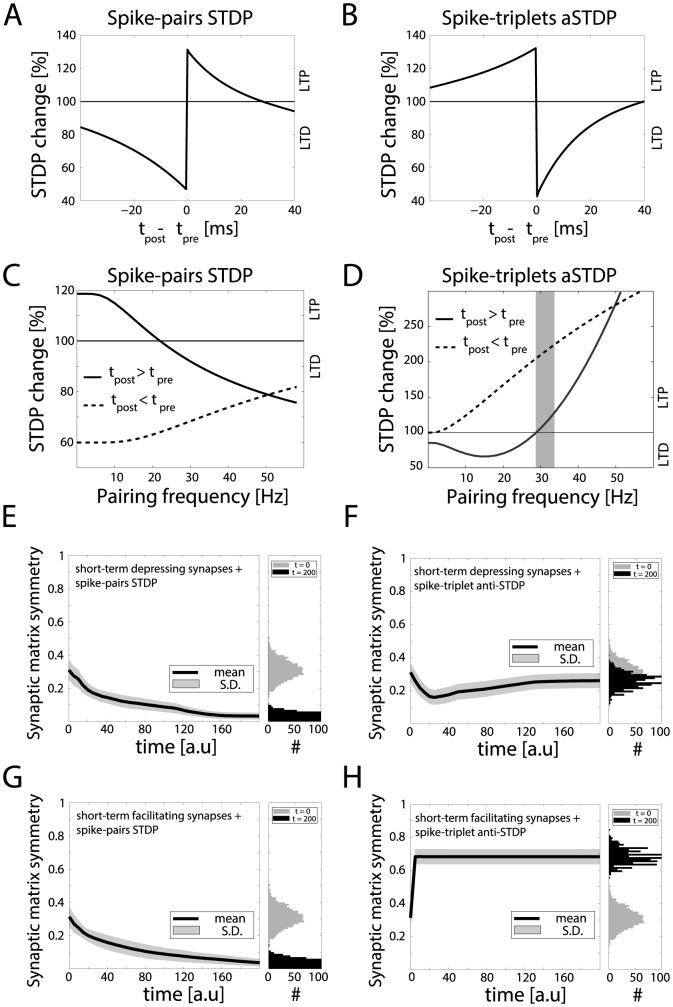
STDP key features for motif emergence. The pair-based STDP model, with temporal window shown in panel **A** and matching exactly Fig. 2 A, exhibits a different frequency dependency (panel **C**) than the triplet-based STDP model (Fig. 2 B). Modifying the triplet-based STDP parameters to *ad hoc* invert its temporal window (e.g., as in anti-STDP, panel **B**, compare to panel **A**), yet leaves its frequency dependency and the LTD-reversal (grey shading) unchanged (panel **D**). Repeating the study of Fig. 2 with these two modified models, we find that (i) the pair-based STDP fails to account for motif emergence (panels **E**, **G**, compare to Fig. 2), while (ii) anti-STDP succeeds (panels **F**, **H**, compare to Fig. 2).

We then confirm the minimal set of long-term synaptic plasticity features sufficient for motif emergence, by performing additional negative and positive control simulations (Fig. 5). We consider the pair-based STDP model [24], [31] and adjust its parameters to get an identical spike-timing dependency to the one predicted by the STDP model we used (i.e., compare Fig. 5 A to Fig. 2 A). Under these conditions, the resulting spike-frequency dependency is completely different (i.e., compare Fig. 5 C to Fig. 2 B), showing no reversal of LTD into LTP at high frequencies.

On the contrary, by making minimal changes to the equations and parameters of the STDP model we used (see Methods and Supplementary Information, Text S1), it is possible to *ad hoc* reverse the temporal dependency of STDP, e.g., as observed experimentally for anti-STDP (aSTDP) [32], while leaving the spike-frequency dependence roughly intact (i.e., compare Figs. 5 B, D to Figs. 2 A, B). This case still features the reversal of LTD into LTP at high frequencies. We note that this modified aSTDP “triplet rule” serves only as a positive control and it is not meant to provide an accurate description of aSTDP, since more data are needed to access its firing rate dependence.

Panels E–H of Fig. 5 repeat the simulations of Fig. 2, and demonstrate that only when the realistic frequency dependence of STDP [22] is present, the heterogeneity in the network firing rates leads to the emergence of asymmetric connectivity motifs (compare Figs. 5 E, G or Figs. 5 F, H to Figs. 2 C, D). We therefore conclude that all long-term plasticity models that capture the temporal nature of the STDP at low frequencies and the LTD to LTP reversal at high frequencies (eg. [14], [33]) would also reproduce our results.

#### Large microscopic simulations of homogenous networks

We further confirm our results by means of numerical simulations of larger recurrent networks, composed of 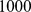 IF neurons.

**Figure 6:**
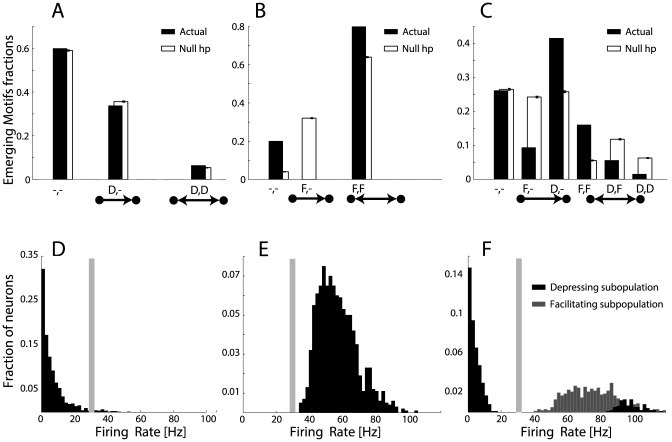
Results from numerical simulations of large recurrent networks of model neurons with short- and long-term plasticity. Homogeneous and heterogeneous recurrent networks made of 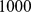 Integrate-and-Fire model neurons were numerically simulated, under identical conditions. Panel **A** shows the comparison of the emergence of weak or no connectivity pairs (indicated as “-, -”), of unidirectional strong connectivity pairs (“
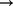
”, “D, -”), and of reciprocal strong connectivity pairs (“
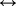
”, “D, D”) for a homogeneous network of neurons connected by depressing synapses: strong unidirectional depressing connections significantly outnumber reciprocal depressing ones. The fractions of emerged motifs (black) is significantly different than the null hypothesis (white) of random motifs occurrence. Panel **B** repeats this quantification for a homogeneous network with facilitating synapses: strong connections are found only on reciprocal connectivity pairs (“
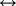
”, “F, F”) and all emerging motifs are non-random. Panel **C** repeats the same quantification for a heterogeneous network with both short-term facilitating and depressing synapses. Emerging motifs display highly non-random features and confirm that reciprocal facilitatory motifs (“
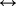
”, “F, F”) outnumber unidirectional facilitatory motifs (“
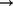
”, “F, -”), and that unidirectional depressing motifs (“
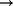
”, “D,-”) outnumber reciprocal depressing motifs (“
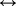
”, “D, D”). Panels **D–F** display the steady-state firing rate distributions, corresponding to homogeneous depressing, homogeneous facilitating, and heterogeneous networks respectively. The plots confirm that heterogeneity in connectivity motifs is accompanied by bimodal firing rates above and below the *critical* frequency, represented here by a grey shading.

In order to increase the biological realism, in these simulations we also introduced fluctuating random inputs to each neuron, mimicking irregular background synaptic activity (see [34], [35] and the Methods). Each neuron thus receives an uncorrelated noisy current, as well as a periodic wave-like stimuli as in the toy model. As in Fig. 2, SD and STDP shape the maximal synaptic efficacies so that unidirectional depressing connections significantly outnumber the reciprocal depressing connections, while facilitating reciprocal connections prevail on unidirectional facilitating connections. Figures 6 A, B display the count of the occurrence of unidirectional *versus* reciprocal connectivity motifs.

Figures 6 D–E show the heterogeneous distributions of the firing rates of the two networks: for the same external input, networks of model neurons with homogeneous depressing short-term plastic synapses fire at low rates, while networks with homogeneous facilitating synapses fire at higher rates. The symmetry index 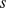, computed after a very long simulation run, results in a value of 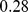 for the depressing network and of 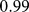 for the facilitatory network.

### Heterogeneous microcircuits

**Figure 7:**
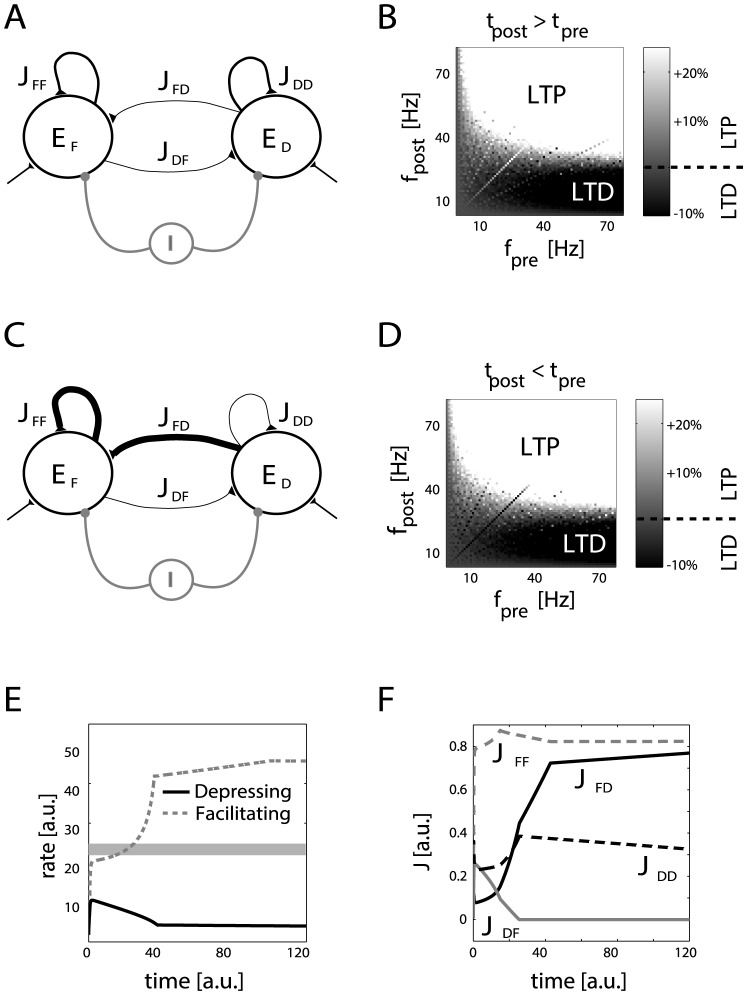
Mean-field simulation of a heterogeneous network with short- and long-term plasticity. The firing rate evolution of a heterogenous recurrent network, including both short-term facilitating and depressing synapses (**A**), was estimated by numerically solving the corresponding mean-field equations. The average synaptic efficacies among and across populations, indicated by 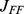, 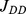, 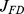, and 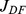, undergo long-term modification. Panels **B, D** show the long-term changes of an isolated synapse (decoupled from recurrent interactions) depending on pre- and postsynaptic spike timing (i.e., 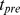, 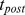) and frequencies (i.e., 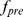, 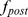). When 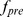 and 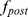 are varied independently, long-term potentiation (LTP) and depression (LTD) emerge as in associative Hebbian plasticity. This suggests that 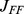 and 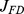 will become significantly stronger than 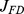 and 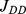 (**C**) and that such a configuration will be retained indefinitely. This was confirmed by simulations (**E–F**) plotting the temporal evolution of the firing rates 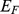 (black trace) and 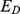 (grey trace), and of the mean synaptic efficacies 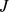 (**F**). The heterogeneity occurs by separation of emerging firing rates (Fig. 4), as emphasized by the grey shading. Parameters: 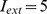, 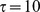
msec, with initial conditions for the maximal synaptic efficacies 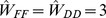, and 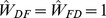. Please note that without loss of generality we fix 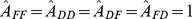.

Here, we study the more general case of a heterogeneous model network (Fig. 7 A) with two subpopulations: one containing neurons connected by facilitating synapses, and the other containing neurons connected by depressing synapses. We show that our results on the emergence of connectivity motifs still hold, as long as the connections across the heterogenous populations are initially weak, preventing them form affecting each other's firing rates.

Synapses established within neuronal pairs that belong to the same subpopulation, share, by definition, the same SD properties, i.e., short-term depressing or short-term facilitating, but not both simultaneously. On the contrary, synapses established within neuronal pairs that belong to distinct subpopulations have, by definition, heterogeneous SD properties. Thus a total of five categories of connectivity motifs are possible in this network: facilitatory reciprocal motifs, depressing reciprocal motifs, facilitatory unidirectional motifs, depressing unidirectional motifs, and reciprocal motifs with both facilitation and depression. For the first four categories, experiments support strong non-random distribution [4], [13]. For the case of reciprocal motifs with both facilitation and depression, no extensive experimental information has been published. Our results suggest that non-random occurrences of the first four categories arise from SD-STDP interactions, in qualitative agreement with the experiments. We also further predict that the last category should be largely underrepresented, compared to chance level.

#### Spike-timing and associative Hebbian plasticity in heterogenous networks

We first examine the impact of STDP in a simplified two-neuron system, with one neuron projecting to the other via a single synapse. Figures 7 B, D show the long-term change in PSP amplitude as a function of the pre- and postsynaptic firing rates, at that single synapse. Since in a heterogeneous network the mean pre- and postsynaptic firing frequencies may differ from each other, we swept the firing frequencies of the two neurons throughout all the possible combinations, within a realistic range (i.e., 0–70 Hz). We studied two cases: each presynaptic spike precedes the postsynaptic spike by 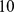 msec, i.e., 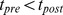, or vice versa, i.e., 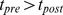. We emphasize that only synapses established within neuronal pairs that belong to distinct subpopulations can experience heterogeneous pre- and postsynaptic firing rates. In this case, however, the impact of spike-timing information becomes negligible, as soon as pre- and postsynaptic neurons fire at different incommensurable frequencies. In the small minority of cases where this is not true, pre- and postsynaptic frequencies are (sub)multiples of each other, and a transient synchronization of spike times occurs periodically. In these circumstances, the timing information has a specific impact, as revealed graphically by the bright or dark straight lines in the plots of Figs. 7 B, D. In all other cases, the overall plasticity profiles reflect the conventional associative Hebbian LTP/LTD and its consequences [30], [36], [37]. This illustration captures the essence of Eq. 2, i.e., the firing frequency of the postsynaptic neuron determines whether the synapse will be potentiated or depressed. Therefore, connections to neurons firing with high frequencies will be strengthened, while connections to neurons with low firing frequencies will be weakened.

#### Mean-field analysis of heterogenous microcircuits

Intuitively, the heterogeneous population of Fig. 7 A can lead to the emergence of realistic connectivity motifs. To illustrate this point, we first ignore that subpopulations might interfere with each other's firing rates: the facilitating and depressing subnetworks are still characterized by higher or lower firing rates, respectively, as previously presented for homogeneous networks. Then, the LTP/LTD maps of Figs. 7 B, D suggest that if such an initial asymmetry of firing rates emerges then it will be maintained indefinitely. The connections 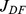 will be in fact increasingly weakened while the connections 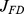 strengthen. The resulting configuration, sketched in Fig. 7 C, is thus stable. We tested and confirmed this statement under the mean-field hypothesis, by numerically simulating the full dynamics of Eqs. 12, 14, and 15, in addition to computing their equilibria. Figures 7 E, F display the mean firing rates of each subnetwork, and the time course of the mean synaptic efficacies. We remind the reader that the maximal synaptic efficacies act on the mean synaptic efficacies as scaling factors, hence a weaker value of 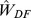 corresponds to a weaker value of 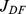. However, the desirable configuration emerges only when the maximal efficacies across populations, i.e., 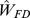 and 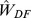, are initialized to slightly weaker values than the maximum intra-population efficacies, i.e., 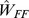 and 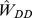. This prevents the facilitating subpopulation to transiently, but irreversibly, drive above 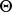 the activity of the depressing subpopulation. In Text S1, we partially relax this condition showing how weak synaptic connections across subpopulations may still evolve from fully homogeneous initial couplings.

#### Large microscopic simulations of heterogenous networks

We further evaluate our results by numerical simulations of a large heterogenous network, as in Fig. 6 C (see Methods). These simulations involve 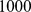 Integrate-and-Fire units, subdivided in two subpopulations of equal size, with a structural connectivity set to approximately 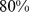 of all possible connections, as in the homogenous case.

As in the mean-field model, the scaling factors of the maximal synaptic efficacy 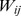 across populations are initialized to weaker values than the intra-population terms. Each neuron receives an uncorrelated background noisy current as well as periodic wave-like stimuli, similar to the homogeneous case. As indicated by Fig. 6 F, the firing rate distributions is bimodal: neurons in the subnetwork of depressing short-term plastic synapses fire at generally low rates, while neurons in the subnetwork of facilitating synapses fire at higher rates. The location of the *critical* firing frequency for the STDP is represented again as a grey shaded area.

Results in Fig. 6 C show all the possible synaptic combinations. Qualitatively similar to the data of Wang *et al.* (2006), reciprocal motifs are significantly co-expressed with facilitatory synapses and unidirectional motifs with depressing synapses. The actual motif counts are compared to the null hypothesis of having statistical independence between the connection occurrence within a pair of neurons, estimated at a 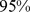 confidence interval upon the same hypothesis of *Bernoulli* repeated, independent, elementary events. The frequency 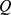 of observing a connection between two neurons, regardless of its SD properties, is first estimated by direct inspection of the connectivity matrix 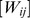. Then the conditional occurrence frequencies of a facilitatory synapse 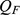 and of a depressing synapse 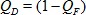 are computed, given that a connection exist between two neurons. The null hypothesis for each possible combination is given by standard probability calculus, under the hypothesis of independence of identical events. For example, the occurrence frequency of observing by chance no connections within a neuronal pair is 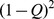, while the occurrence of observing by chance a reciprocal motifs with mixed depressing and facilitating properties is 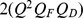.

Finally, as the symmetry index 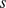 was computed over a very long simulation run, it resulted in a value of 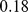 for the depressing subnetwork and of 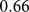 for the facilitation subnetwork.

### Microcircuits with overlapping SD properties

In the heterogeneous network of Fig. 7, as well as in the homogeneous networks, we make the assumption that the SD profile is determined primarily by the identity of the projecting neuron. Therefore the same neuron always establishes short-term depressing or short-term facilitating synapses with its target. This has been experimentally found in the olfactory, visual, and somatosensory cortices as well as in other brain areas [38]–[41]. Nonetheless, the assumption on the projection-cell specificity can be removed in order to theoretically explore the impact of SD heterogeneity across distinct synaptic connections, established by the same presynaptic neuron [42].

**Figure 8:**
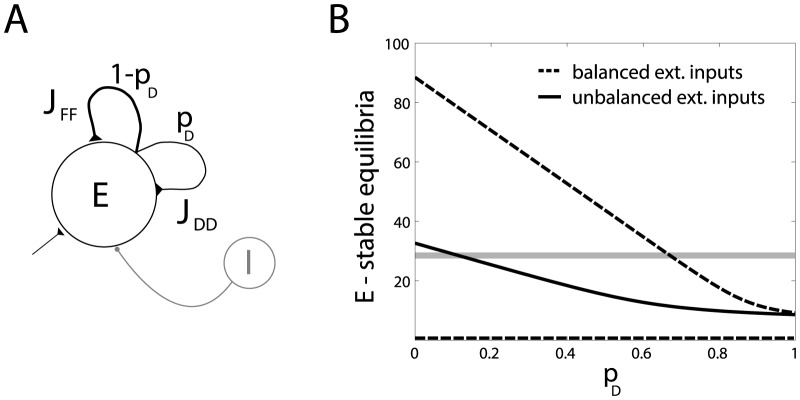
Mean-field analysis of firing rate equilibria, in homogeneous networks with overlapping short-term synaptic properties and no long-term plasticity. Panel **A** represents the sketch of a recurrent network where a clear segregation between subpopulations of depressing- or facilitating-only synapses does not occur. A neuron has a probability 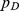 of connecting to its postsynaptic target by a depressing synapse, and 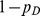 of connecting to its target by a facilitating synapticapse. Panel **B** plots the location of the equilibria of the firing rate 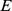, under distinct external inputs conditions and for increasing values of 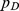. For the same parameters of Fig. 4, stable equilibria move as a function of 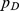, taking intermediate values between the two extreme cases, i.e., 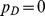 and 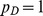; compare to panels **D–F** of Figs. 4 .

We assume that a generic neuron has a certain probability 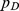 of establishing a short-term depressing synapse with a target neuron, and a probability 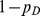 of establishing a short-term facilitating synapse with another one. In this case, individual neurons are still indistinguishable (Fig. 8 A). For small values of 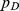, the emerging firing rates approximate those of a network of facilitating synapses, while for large values of 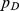 the firing rates behave as for a network of depressing synapses. In other words, the mixed networks behave dynamically as an intermediate case between two extremes. This result is quantified in Fig. 8 B, where the location of the stable equilibrium points has been computed under the mean-field hypotheses and plotted as a function of 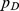, for different external inputs regimes. The qualitative location of the *critical* firing frequency for the STDP is represented as a grey shaded area. As an explicit consequence of the lack of any structure (i.e., compare Fig. 8 A with Fig. 7 A), STDP fails to discriminate individual connections within the network, but rather shapes them as reciprocal or as unidirectional motifs, depending on the particular choice of 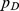. For a simple mechanism of how the desirable structure may evolve please see Text S1.

## Discussion

The impact of long-term synaptic plasticity in recurrent networks of spiking model neurons has been studied earlier in the context of stimulus-driven dynamical attractors of network activity representing working-memory states [30]. Within the same aims, the interactions between long-term plasticity and SD were also partly explored, both in numerical simulations and in mean-field descriptions [43]. In this work, we focus on a specific long-term plasticity mechanism (STDP) [9] previously reported to lead to the emergence of network structure [44]–[47] and connectivity motifs [14], [48]. To the best of our knowledge, the interaction of SD and STDP has not been previously considered as key element for the emergence of non-random network connectivity.

Our modeling results indicate that time- and frequency-dependent STDP mechanisms may be responsible for the observation that excitatory model neurons connected by short-term facilitating synapses are more likely to form reciprocal connections, while model neurons connected by short-term depressing synapses are more likely to form unidirectional connections. More specifically:

1. The internally generated firing rates in model networks with facilitating connections are higher than in networks with depressing connections, under identical background/external inputs;
2. Neurons, participating in such an internally generated activity, are likely to form bidirectional connections with each others when firing at sufficiently high rates, reflecting the “Hebbian” mode of STDP; neurons firing at low rates are likely to form unidirectional connections, reflecting the temporally asymmetric “pre-post” temporal mode of STDP;
3. Once formed, these connectivity motifs persist through the internally-generated firing activity of the network, from which the motifs emerged;
4. Externally generated inputs, strongly depolarizing or strongly hyperpolarizing individual neurons, when prevailing over internally generated activity, may lead to opposite motif emergence.

Our results suggest a mechanism that should be considered when explaining the features of connectivity motif emergence in the cortical pyramidal microcircuitry. Preliminary data collected in the olfactory bulb reveal the same trend, therefore our framework might point to a common principle for synaptic wiring in different brain areas. For instance, long-term and short-term plasticity has been experimentally found among olfactory mitral cells [13], [49]. STDP was reported in the rodent and insect olfactory systems [50], [51], and considered as a mechanism that explains decorrelation of sensory information [52] in mitral cells.

The major difference in SD properties, which accounts for motif emergence, is the heterogeneity of the time constant representing the short-term depression recovery 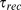. In this respect, our results and conclusions would be qualitatively unchanged by replacing facilitating synaptic properties with linear, i.e., non-depressing, properties. Along these lines, we predict that the value of 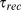, in a pair of connected neurons should be inversely correlated to the occurrence frequency of reciprocal motifs.

A key simplifying hypothesis of our work is that STDP scales only the SD parameter 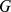, i.e., the PSC amplitude, leaving the parameter 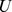, which represents the maximal usage of resources, unaltered [53]. This choice is biologically consistent but not representative of all cortical areas [54]. Although the debate on pre- and postsynaptic expression of plasticity is fierce, our choice of SD and STDP interaction is in part an arbitrary hypothesis and in part supported by experimental evidences [55]. This choice serves as a first solid ground for our conclusions. Enabling STDP to modify the parameter 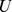 would have partly altered in an activity-dependent manner the SD profile of a synapse. This would have made isolating SD contribution more complex, and relating our findings to previous theoretical works [14], [44]–[47] less straightforward. The model itself is purely phenomenological, and does not capture biophysical details, but rather the interaction of SD and STDP via a set of variables locally known to the synapse. It does maintain, however, the desirable compatibility with experimental data. In addition, detailed information on the STDP mechanisms at excitatory facilitatory synapses in prefrontal cortex are currently scarce [4]. While more efforts, both experimental and theoretical, should be undoubtedly devoted in these directions, the hypothesis of scaling 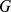 and not 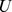 remains a simplifying assumption, in view of the lack of a systematic understanding on how STDP affects all the parameters of the SD model [56].

Our proposed mechanism for non-random pattern emergence is based on the sole interactions between STDP and SD. Obviously, it is unlikely that these mechanisms operate independently of other synaptic phenomena. Homeostatic plasticity could for instance continuously rescale synaptic efficacy and make SD heterogeneities less predominant in determining connectivity motifs. In the lack of *a priori* experimental information, we chose the maximal synaptic efficacy to be the same for all the microcircuits we examined, in order to ensure a fair comparison. With all its limitations, our proposal may still provide a simple working hypothesis on one component underlying the emergence of connectivity, linked to short-term synaptic dynamics, along the same lines of the theory proposed by Clopath *et al.* (2010). Its validity could be challenged by experiments that interfere and probe the emergent firing activity, e.g., in local *in vitro* cultured microcircuits with known synaptic properties [57].

In the context of the motifs, we have also limited our study to a stereotypical external signal, with the view to demonstrate that under identical external stimuli, facilitating and depressing (sub-)networks will evolve different structures. To this end, it would be of interest to investigate how such motifs would emerge while learning a specific task, in the context for instance, of reinforcement- [58], [59] or unsupervised-learning [15].

Our framework might be also useful for investigating further structure–function relationships at the subcellular level, by altering the synaptic machinery, or by employing (future) genetically-encoded fluorescent reporters of synaptic efficacy and dynamics. The use of optogenetics and genetically-encoded neuronal voltage and calcium sensors may lead to experimental validation or falsification of our hypothesis, which might directly contribute to understand short- and long-term plasticity interactions.

We emphasize that our theory refers only to one of many possible, perhaps competing, mechanisms that contribute to stereotypical motif emergence. Alternative explanations and a causal demonstration of the key ideas we suggest remain to be provided. It might be of interest exploring to which extent developmental changes in SD, such as the switch from depression into facilitation at synapses between layer 5 pyramidal neocortical neurons [40], occurring after postnatal day (P) 22, are mirrored by changes in motif statistics. For marginal pairwise probability of connection, Song *et al.* (2005) [2] report no significant dependence on age, but provide no systematic characterization of motif statistics beyond P20.

It may be also possible to attempt a chronic manipulation of the firing rates of neuron (sub)populations, by pharmacologically altering synaptic profiles, e.g., modulating postsynaptic receptor desensitisation, changing the presynaptic probability release, or interfering with neurotransmitter recycling. As future directions, more complex heterogeneous anatomical architectures and single-cell properties should be incorporated within the same computational modeling framework. Very specific, non-random initial architectures, e.g., small-world and scale-free [60], could be explored, extending our results towards other aspects that determine reciprocal or unidirectional motifs, possibly beyond the firing levels and towards, for instance, the density of hub nodes, ranking orders or heavy tails in distribution of neighbours.

Finally, we underline the great value of physiological information that may accompany the anatomical connectivity. These complementary data-sets contain precious statistical information regarding the expression of microcircuit motifs [2], [3]. We believe that computational modeling is, in this context, a very powerful tool to explore additional hypotheses and challenge further theories.

## Materials and Methods

We study and numerically simulate networks of spiking model neurons [21], connected via plastic, *current-based*, excitatory synapses [6], [14]–[16], [19], [20]. We conventionally distinguish between “strong” and “weak” connections, and provide a simple measure to quantify the occurrence of strong pairwise motifs in our model networks. We finally examine a Wilson–Cowan firing rate model that is helpful for the interpretation of the numerical results. The values of all model parameters are indicated in Table 1, while the full numerical implementation in MATLAB (The Mathworks, Natick, USA) and in ANSI-C is available from ModelDB [61] at http://senselab.med.yale.edu/modeldb via accession number 150211.

### Neuron model

The network is composed of identical adaptive exponential Integrate-and-Fire (IF) neurons [21], each described by a membrane potential 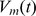 and by a spike-frequency adaptation variable 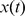 [62]. Below a threshold 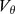, 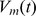 satisfies the charge-balance equation
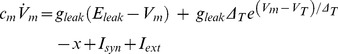
(3)where 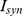 is the synaptic input from other neurons and 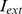 the external (background) input currents. When a spike occurs, i.e., 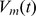 crosses 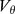, 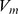 is reset to a 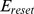.

The spike-frequency adaptation variable 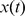 evolves as
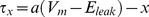
(4)When a spike occurs, 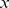 evolves as 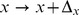. The numerical integration of Eq. 3 is suspended for a period of time 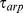 following each spike, to mimic absolute refractoriness, during which 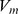 remains “clamped” at 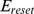. The model details are not essential to our conclusions (not shown).

### External (background) inputs

Each neuron, identified by an index 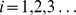, receives a time varying input 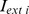 according to the following protocols.

#### Toy Network

(Fig. 1 B, 10 neurons) 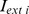 consists of a 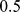 nA constant current, as well as periodically repeating 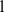 nA square pulses. Pulses occur as in a traveling wave of activity, which moves every 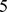 msec from one unit, e.g., the 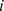
th neuron, to its neighbour, i.e., the 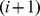
th neuron. In space, each pulse is delivered in turn to all neurons as an extremely narrow bell-shaped profile, with unitary peak amplitude and standard deviation of 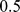, resulting in neighbouring neurons being only weakly stimulated simultaneously. Each pulse is of sufficient amplitude to elicit firing in the unit where the bell-shaped profile is centred on, e.g., the 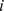
th unit.

#### Large Network

(Fig. 6, 1000 neurons) 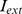 is as in the toy network with the addition of a (spatially) uncorrelated gaussian noisy term [29], with mean 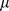, standard deviation 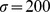 pA, and autocorrelation time length 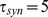 msec. Parameter 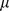 is drawn randomly for each neuron of the network, before launching the simulation, using a normal distribution with mean 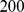 pA and unitary coefficient of variation. The noisy current mimics asynchronous synaptic inputs from (not explicitly modeled) background populations [35], [63].

### Internal (synaptic) inputs

Neurons connect to each other according to a fixed wiring matrix 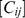, which indicates whether the 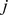
th neuron projects to the 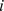
th neuron, i.e., 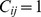, or not, i.e., 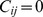. The matrix 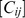 is obtained from an all-to-all connectivity without autapses (i.e., 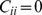), upon randomly pruning *approximately* 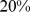 of its elements (see e.g., Fig. 1 B). This is performed uniquely to introduce a more realistic variability in firing across neurons. A more substantial reduction of the structural connectivity does not affect our conclusion qualitatively, although it downscales the number of plastic synapses available for further statistical analysis.

The 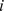
th neuron receives at any time a total synaptic current 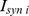, described as
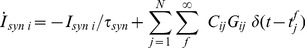
(5)where 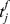 represents the occurrence time of the 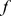
th spike emitted by the 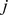
th presynaptic neuron, and where 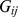 is the peak amplitude of the elementary postsynaptic current (PSC), corresponding to the activation of the synapse by the presynaptic 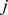
th neuron. The Dirac delta function 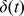 is employed to represent the occurrence of a presynaptic action potential. Thus Eq. 5 models individual PSCs with instantaneous rise time and exponential decay [64]. In terms of implementation, this implies that in the lack of any presynaptic activity, 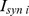 decays exponentially to zero with a time constant 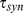 and that, as a presynaptic spike is fired, 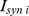 evolves as 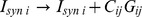.

### Frequency-dependent short-term synaptic dynamics (SD)


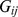
 defines the amplitude of the PSC from presynaptic neuron 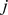
th to postsynaptic neuron 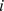
th and is proportional to the amount of used resources for neurotransmission 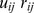 and to their maximal availability 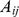, i.e., 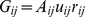.

Frequency-dependent short-term synaptic dynamics are described by
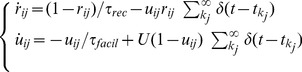
(6)The above equations, with a different set of parameter values, have been shown to capture depressing or facilitating synapses [6] and are widely employed by the community.

For the sake of notation, indexes have been dropped from 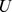, 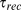, and 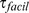 in Eqs. 6, although each synapse has its own parameters (see Table 1). In terms of implementation, Eqs. 6 reduce to the following update rules: (i) when no spike is fired by the presynaptic neuron 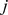, 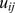 and 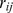 recover exponentially to their resting values, 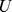 and 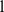, respectively; (ii) as a presynaptic spike occurs, 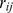 is reduced as 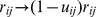, while 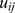 is increased as 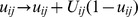. The impact of short-term plasticity of PSCs amplitude has been exemplified in Figs. 1 C, F.

### Spike-timing dependent long-term plasticity (STDP)

We further extend the description of PSCs (Eqs. 5–6) by an additional scaling factor 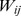, which incorporates STDP [9], see also [65]:
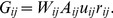
(7)

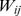
 changes on timescales longer than 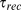 and 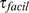 according to the correlated activity of both pre- and postsynaptic neurons, closely following the model proposed by [15].

Briefly, each neuron is complemented by four variables, i.e., 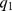, 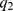, 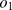, 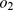, which act as running estimates of its firing rate [27], over distinct time scales, i.e., 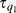, 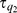, 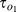, 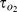. In the lack of any activity of the 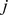
th neuron, those variables exponentially relax to zero:
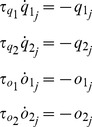
(8)while each time the neuron fires, they are increased by a unit:
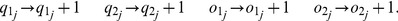
(9)


This enables a compact implementation of STDP: as the 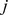
th neuron fires, over all indexes 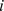


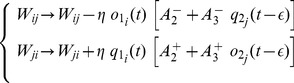
(10)since the 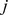
th neuron is presynaptic to all connected 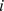
th neurons, and postsynaptic to all connected 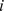
th neurons, respectively. Numerically, the evaluation of 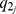 and 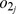 is performed just before the 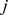
th neuron spikes, as indicated by the infinitesimal time-advance notation of 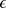. When no spike occurs, 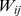 retains indefinitely its value. As in the large majority of STDP implementations, the value of 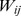 is further bounded in 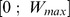 and, unless otherwise stated, randomly initialized prior to the start of each simulation.

To graphically illustrate this model, we isolate the impact on synaptic efficacy of Eqs. 8, 9, 10 in a two-neuron system, with one neuron projecting to the other via a single synapse. We simulate the long-term change in PSP amplitude at that synapse according to the standard STDP protocol (see Fig. 2 A), imposing (i) each presynaptic spike precedes the postsynaptic spike by 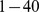 msec, i.e., 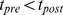 (pre-post protocol) or (ii) vice versa, i.e., 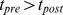 (post-pre protocol). We further apply a frequency STDP protocol (see Fig. 2 B and [15]), imposing 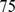 pre-post spike pairing events, evoked at regular increasing pairing frequency. We study two cases: (i) each presynaptic spike precedes the postsynaptic spike by 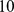 msec, i.e., 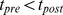, or (ii) vice versa, i.e., 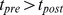. The latter reveals the two regimes, depending on spike-timing and on spike-frequency (separated by the grey shading).

Due to the generality of the formulation of this model, we can easily modify it to produce different STDP curves. By setting 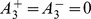, and 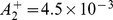 in Eq. 10 and slightly modifying Eq. 9, one can reproduce the pair-based STDP model [24], [31]. By inverting signs and swapping the values 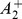, 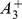, 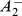, and 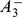 in the same equations, it is possible to *ad hoc* reverse the temporal dependency of STDP, as observed experimentally for anti-STDP (aSTDP) [32], while leaving the frequency dependence roughly intact. We have used both these forms to identify the minimal requirements of an STDP model in the context of the motifs formation that we study.

### Convention on “strong” and “weak” connections and motifs symmetry index

In this study, we focus on the appearance or disappearance of a strong connection between two neurons, but only for units that are anatomically connected, i.e., 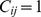. For the sake of comparison, we adopted the framework of Clopath *et al.* (2010), where the activity-dependent appearance or disappearance of a connection conventionally occurs in terms of a competition among “strong” links in a “sea” of weak synapses. As in their paper, we adopt the convention of identifying as “strong” those connections whose factor 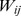 is above the 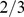 of its upper bound 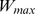.

With such a definition, we measure the average motifs reciprocity by a symmetry index, obtained by counting reciprocal or unidirectional motifs as
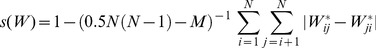
(11)where 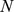 is the size of the matrix 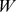, as well as the size of the network (see, e.g., [66] for alternatives). The symmetry index 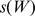 takes values in the range 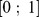 and depends on the average *similarity* between elements of 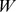 that are on symmetric positions with respect to the diagonal. Following our previous convention, 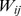 and 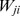 are first normalized and then zero-clipped: 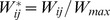 if 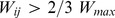, and otherwise 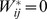. In Eq. 11, 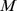 represents the number of null pairs 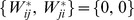 that occur as a consequence of clipping or by initialization and pruning.

Evaluating 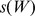 on networks with a majority of unidirectional connections results in values close to 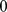 (e.g., Fig. 2 A), while its evaluation on networks with a majority of reciprocal connections results in values close to 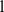 (e.g., Fig. 2 B). For uniform random matrices 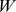, it is possible to calculate the full statistics of 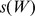 and use it for deriving a significance measure for 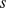 as a 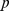-value, being the probability that the value of 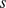 observed in simulations could result by chance.

### Mean-field Network Description

We analyze the firing rate of the IF network through its mean-field dynamical description [18], [27], [67], closely following earlier work [16], [19]. We assume that (i) the network consists of one or more non-overlapping subpopulations (see Figs. 4 A and Fig. 7 A) and that (ii) neurons within each subpopulation share identical synaptic coupling, connectivity, and short-term synaptic plasticity properties, i.e., all depressing or all facilitating, as in Fig. 7 A; see Fig. 8 A for an exception. We also assume that (iii) for each (sub)population, individual neuronal firing occurs as a Poisson point process, with instantaneous mean firing rate 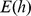, which depends monotonically on the corresponding average input currents 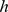. Under these hypotheses, neurons can be distinguished only by the subpopulation they belong to, i.e., depressing D or facilitating F, and their firing rate is indicated as 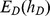 and 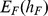. For the case of two subpopulations (Fig. 7 A), 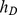 and 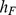 evolve over a characteristic time scale 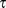 as
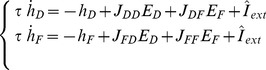
(12)where 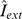 represents the average external input, 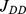 and 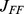 the average synaptic efficacies of recurrent connections within each subpopulation, and 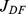 and 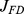 the average synaptic efficacies of connections across subpopulations. On a first approximation, 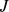 can be considered as the ensemble average over 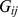 and firing rates 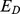 and 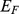 can be computed from 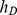 and 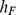 as threshold-linear frequency–current response functions: 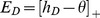 and 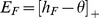, with 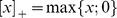 (for alternatives see [68], [69]). 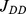, 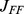, 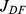, 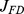 undergo short and long plastic changes. Indicating by 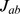 the mean synaptic efficacy between the presynaptic population 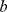 and the postsynaptic population 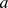, then
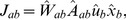
(13)with 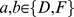. The quantities 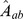 and 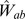 represent the maximal synaptic efficacy and the weighting factor modified by STDP. The quantities 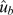 and 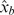 depend only on the presynaptic firing rate 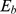, and capture the short-term homosynaptic plasticity in the mean-field version of Eqs. 6 [16]:
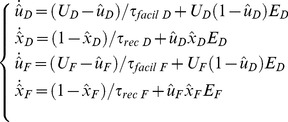
(14)


On longer timescales, controlled by the plasticity rate parameter 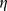, we adopt the mean-field approximation of STDP given in [15]: the factor 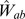 evolves as a function of both presynaptic 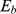 and postsynaptic 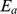 rates:
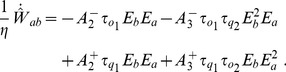
(15)


With parameters 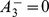 and 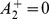, as in our simulations, see Table 1, the above expression can be rewritten as
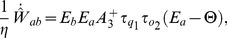
(16)with 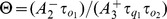. Considering the postsynaptic activity 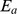 fixed, the stability analysis shows that the point 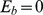 is stable if 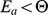 and unstable if 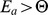. In the latter case, the weight will increase towards its upper bound. Considering the presynaptic activity 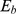 fixed, there are two equilibrium points: 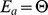, which is unstable, and 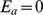, which is stable. Hence, the postsynaptic activity will tend to go to either the maximum possible value or to zero depending on whether initially 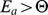 or not. This translates to weights going to their upper bound or to zero.

The mean approximation is derived under the assumption of Poisson distributed presynaptic and postsynaptic firing times. When there is temporal (pre-post) correlation in the external activity, which the model is able to capture, it would lead to unidirectional connections and non-zero postsynaptic activity, see also Text S1.

## Supporting Information

Text S1 **A Supplementary Information (Text S1) accompanies this paper and reviews in detail the basic mean-field analysis of firing rate stability of recurrent networks of model neurons with plastic synapses.** It also explores the impact of recurrent inhibition, presents a viable alternative to heterogeneous initial weight initialization, and a analytical description for the development of unidirectional motif for networks with low firing activity imposed temporally correlated external inputs. Further, it provides the implementation details of the alternative STDP models (i.e., pair-based and anti-STDP), and demonstrates that results presented in the main text are independent on IF model details. It finally provides full statistics of the conventional symmetry index employed in our paper and reveals its sensitivity on the value-clipping threshold.
(PDF)
