# Supplementary material for: Emergence of Connectivity Motifs in Networks of Model Neurons with Short- and Long-Term Plastic Synapses
Source: PLoS One. 2014 Jan 15;9(1):e84626. doi: 10.1371/journal.pone.0084626 (PMC3893143; doi:10.1371/journal.pone.0084626)
Supplement: Text S1 — A Supplementary Information (Text S1) accompanies this paper and reviews in detail the basic mean-field analysis of firing rate stability of recurrent networks of model neurons with plastic synapses. It also explores the impact of recurrent inhibition, presents a viable alternative to heterogeneous initial weight initialization, and a analytical description for the development of unidirectional motif for networks with low firing activity imposed temporally correlated external inputs. Further, it provides the implementation details of the alternative STDP models (i.e., pair-based and anti-STDP), and demonstrates that results presented in the main text are independent on IF model details. It finally provides full statistics of the conventional symmetry index employed in our paper and reveals its sensitivity on the value-clipping threshold. (PDF) [file pone.0084626.s001.pdf]

Supplementary Information

Eleni Vasilaki, Michele Giugliano

This Supplementary Information accompanies the paper entitled *Emergence of Connectivity Motifs in Networks of Model Neurons with Short- and Long-term Plastic Synapses* and reviews in detail the basic mean-field analysis of firing rate stability of recurrent networks of model neurons with plastic synapses. It also explores the impact of recurrent inhibition, presents a viable alternative to heterogeneous initial weight initialization, and a analytical description for the development of unidirectional motif for networks with low firing activity imposed temporally correlated external inputs. Further, it provides the implementation details of the alternative STDP models (i.e., pair-based and anti-STDP), and demonstrates that results presented in the main text are independent on IF model details. It finally provides full statistics of the conventional symmetry index employed in our paper and reveals its sensitivity on the value-clipping threshold.

1 Supplementary Text S1

For the sake of illustration of the standard mathematical techniques employed in the main text, we consider two extreme simplifications of the mean-field description of a recurrent networks with short term plastic synapses (see also [1] and references therein): the predominance of depressing mechanisms or the predominance of facilitatory mechanisms. We analyze these two cases and specifically study the stability of dynamical equilibria, indicating the factors that affect the maximum (stable) firing rate.

Further, we comment on the analysis of the full model of short term synaptic plasticity and comment on the impact of adding recurrent inhibition to our scenario. We then consider and numerically analyze the mean-field description of a mixed population, where a generic neuron may establish simultaneously depressing and facilitating synapses to its targets.

We also examine and partly relax the necessary conditions for a heterogeneous network of two interacting subpopulations to display emergence of connectivity motif. We provide model details for the pair-based STDP as well as for the anti-STDP "triplet" model, discussed in the main text and analytically show that the external temporal simulation protocol employed in our simulations promotes unidirectional connectivity.

Finally, for the case of a random matrix (i.e., as a null hypothesis), we provide some of the statistical properties of the symmetry measure we adopted to quantify the connectivity motif and derive a confidence measure.

1.1 Single population with depressing synapses

The "Mean-field Network Description" of the main text can be simplified under the hypothesis of very fast recovery from facilitation. In what follows, for simplicity we drop

the notation ˆ of the main text for all mean-field equations. We assume that τ_{facil} is very small and that $u = U$ does not vary in time as a consequence. In this case, only short term depression is modifying the synaptic efficacy, so that the mean-field firing rate dynamics of a large recurrent network of excitatory neurons can be described by a system of two dynamical equations [2]:

$$\begin{cases} \tau \dot{h} = -h + AUxE + I_{ext} \\ \dot{x} = (1-x)/\tau_{rec} - UxE \end{cases} \tag{S1}$$

These are coupled non-linear differential equations that can be analyzed by standard methods of dynamical systems theory [3]. We will consider here the derivation of the equilibria for h and x and indicate how their stability was assessed. By definition of equilibrium, $\dot{h} = 0$ and $\dot{x} = 0$ for those points (also called *fixed* points). Substituting these conditions in Eqs. S1, we obtain two non-linear algebraic equations,

$$\begin{cases} h = AUxE + I_{ext} \\ x = 1/(1 + U\tau_{rec}E) \end{cases} \tag{S2}$$

Using the second equation to replace x as it appears in the first, we get an implicit equation in the unknown h:

$$h = AUE/(1 + U\tau_{rec}E) + I_{ext} \tag{S3}$$

Equation S3 can be solved numerically (e.g., by the Newton-Raphson method [4]), given a specific set of parameters A, U, τ_{rec}, and I_{ext}. Alternatively, its solution(s) can be interpreted graphically as the intersection(s) between two functions of h, $F_1(h)$ and $F_2(h)$ in the cartesian plane,

$$\begin{cases} F_1(h) = h \\ F_2(h) = AUE/(1 + U\tau_{rec}E) + I_{ext} \end{cases} \tag{S4}$$

with $F_1(h)$ is the unitary slope line (see Fig. S1). Retaining the graphical interpretation, it is possible to appreciate intuitively the existence of the equilibrium points and their dependence on the parameters, as explored in Fig. S1. To this aim it is useful, prior to plotting $F_2(h)$, to analytically determine some of its mathematical properties such as the asymptotic limits and derivatives.

We observe that, by definition of $E = [\alpha(h-\theta)]_+$, $F_2(h)$ is zero for values of h lower than θ, $h \leq \theta$. As $h \to +\infty$, $F_2(h)$ tends to an horizontal positive asymptote, occurring at $(A\tau_{rec}^{-1} + I_{ext})$. For values of h larger than θ, the first derivative of $F_2(h)$ is always positive, indicating that the function is monotonically increasing. In the same range of h, the second derivative is instead always negative, therefore indicating that the function

is convex. Moreover, the tangent line to $F_2(h)$ at $h = \theta$ is the steepest of all the tangents to subsequent points ($h > \theta$).

$$\begin{cases} \dot{F}_2(h) = \alpha AU / \left[1 + \alpha \tau_{rec} U \left(h - \theta\right)\right]^2 \\ \ddot{F}_2(h) = -2\alpha^2 \tau_{rec} AU^2 / \left[1 + \alpha \tau_{rec} U \left(h - \theta\right)\right]^3 \end{cases} \tag{S5}$$

The value of the slope of the tangent line at $F_2(h)$ at $h = \theta$ is particularly informative when drawing $F_2(h)$, since at any coordinates $h > \theta$ all the other tangent lines are by definition less steep than it. This maximal slope ($\dot{F}_2(\theta) = \alpha AU$) can then be used as a necessary condition for the intersections between $F_2(h)$ and the unitary slope line, at least when $I_{ext} \leq \theta$.

When $I_{ext} \leq 0$, there is always an intersection between $F_2(h)$ and $F_1(h)$ at $h = I_{ext}$. This is a stable equilibrium point (i.e., see below for the discussion of the stability). If $\alpha AU \leq 1$, there will be for $I_{ext} \leq 0$ no other intersections, since $F_1(h) = h$ has unitary slope itself. Given the necessary condition $\alpha AU > 1$, there exist a minimal value for those parameters above which other two intersections (i.e., one stable and one unstable) with the unitary slope lines occur (compare Figs. S1A,B, and C, which were obtained for increasing values of A).

Determining analytically such values requires imposing the condition where the unitary slope line becomes tangent to $F_2(h)$. Mathematically this can be expressed by stating that when $I_{ext} \leq \theta$ there is a specific (intersection) point $h_0 > \theta$ between $F_1(h)$ and $F_2(h)$. By definition, this point lays on the unitary slope line $F_2(h_0) = h_0$, with $\dot{F}_2(h_0) = 1$, i.e., $F_2(h)$ has a unitary first derivative at h_0 (see Fig. S1B).

$$\begin{cases} F_2(h_0) = \alpha AU(h_0 - \theta) / \left(1 + \alpha \tau_{rec} U(h_0 - \theta)\right) + I_{ext} = h_0 \\ \dot{F}_2(h_0) = \alpha AU / \left[1 + \alpha \tau_{rec} U \left(h_0 - \theta\right)\right]^2 = 1 \end{cases} \tag{S6}$$

Simplifying the algebraic manipulations, we note the apparent similarities between the two equations above. We express (part of the) numerator and denominator of the right hand sides of each equations by denoting the common terms as N_0 and D_0, respectively, as it follows

$$\begin{cases} F_2(h_0) = N_0(h_0 - \theta)/D_0 + I_{ext} = h_0 \\ \dot{F}_2(h_0) = N_0/D_0^2 = 1 \end{cases} \tag{S7}$$

Thus, from the second equation we derive $N_0/D_0 = D_0$ and we substitute it in the first, obtaining

$$h_0 = \theta + \sqrt{\left(\theta - I_{ext}\right) / \left(\alpha U \tau_{rec}\right)} \tag{S8}$$

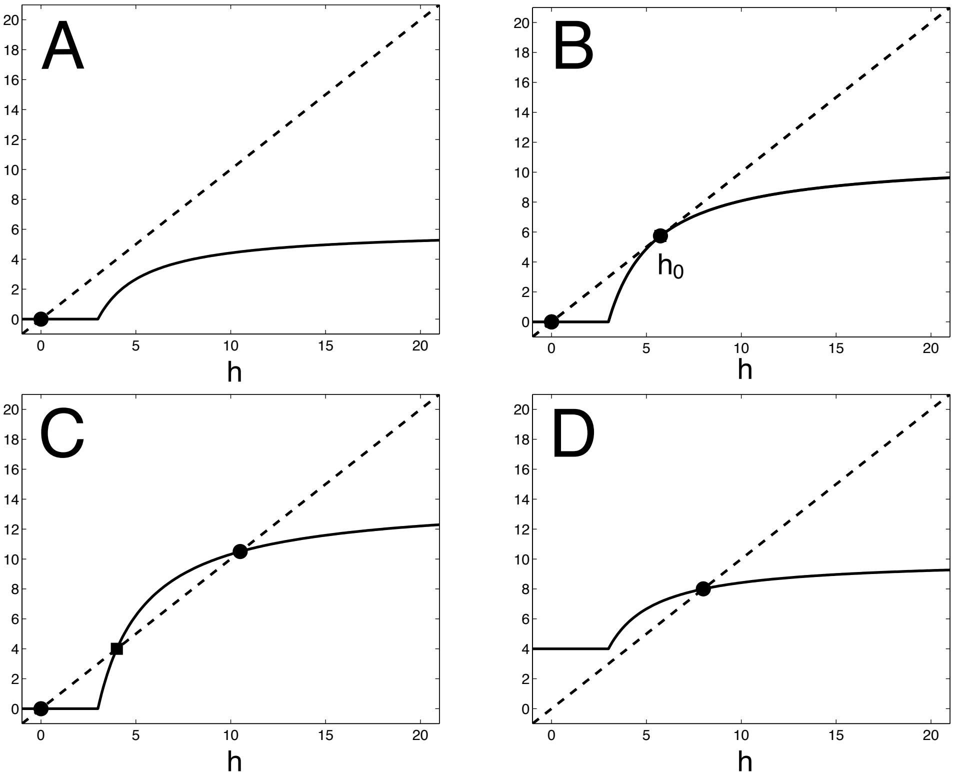

Figure S1. Graphical representation of the function $F_2(h)$ for different values of A and I_{ext}. The function $F_2(h)$ has been plotted for different values of the maximal synaptic efficacy A and external input I_{ext}, resulting in one intersection only (**A**: $A = 3$, $I_{ext} = 0$) , three intersections with two of them coincident with each other (**B**: $A = 5.488$, $I_{ext} = 0$), three distinct intersections (**C**: $A = 7$, $I_{ext} = 0$), and finally one intersection for larger external input (**D**: $A = 3$, $I_{ext} = 4$). The remaining parameters were: $U = 0.8$, $\tau_{rec} = 500$ msec, $\theta = 3$, $\alpha = 1$. The scripts to generate these plots and to carry out asymptotic analysis on the stability of the equilibrium points, see the text, are available online from the ModelDB (accession number 150211).

The above expression is of course only defined when the argument of the square root is positive, which is consistent with our previous hypothesis (i.e., $I_{ext} < \theta$). One can now replace h_0 in the second equation of Eqs. S7 and obtain the corresponding critical value of A associated to the existence of such a (double) intersection (Fig. S1B):

$$A_0 = \left(1 + \sqrt{\alpha U \tau_{rec} \left(\theta - I_{ext}\right)}\right)^2 / \left(\alpha U\right) \tag{S9}$$

In summary, when $I_{ext} \leq \theta$ there is always one (stable) equilibrium at $h = I_{ext}$ and of possibly other two intersections (one stable and one unstable; compare Figs. S1A,B,

and C), depending on the strength of A with respect to A_0. For $I_{ext} > \theta$, the situations changes and the scenario simplifies considerably with only one (stable) intersection for any other choice of the parameters (see Fig. S1D).

The analysis of the stability of the equilibrium points conclude our discussion. Since the dynamical system described Eqs. S1 is non-linear, stability of equilibrium points must be related to the linearized system. The linearization is obtained for each equilibrium point by first-order Taylor expansion of Eqs. S1 around that point. Let's compactly rewrite Eqs. S1 as

$$\begin{cases} \dot{h} = G_1(h,x) = (-h + AUxE + I_{ext})/\tau \\ \dot{x} = G_2(h,x) = (1-x)/\tau_{rec} + UxE \end{cases} \tag{S10}$$

The linearized system around a generic equilibrium point (h_0, x_0) is then

$$\begin{cases} \dot{h} \approx G_1(h_0,x_0) + \partial G_1/\partial h|_{(h_0,x_0)}(h-h_0) + \partial G_1/\partial x|_{(h_0,x_0)}(x-x_0) \\ \dot{x} \approx G_2(h_0,x_0) + \partial G_2/\partial h|_{(h_0,x_0)}(h-h_0) + \partial G_2/\partial x|_{(h_0,x_0)}(x-x_0) \end{cases} \tag{S11}$$

Assessing the stability of the above system reduces to studying the *Jacobian* matrix M:

$$M(h_0,x_0) = \begin{pmatrix} \partial G_1/\partial h & \partial G_1/\partial x \\ \partial G_2/\partial h & \partial G_2/\partial x \end{pmatrix}|_{(h_0,x_0)} \tag{S12}$$

By definition, it is possible to make explicit the Jacobian matrix as

$$M(h_0,x_0) = \begin{pmatrix} (-1+\alpha AUx_0)/\tau & (\alpha AU(h_0-\theta))/\tau \\ -\alpha Ux_0 & -\tau_{rec}^{-1} - \alpha U(h_0-\theta) \end{pmatrix} \tag{S13}$$

In particular, the real part of the two eigenvalues associated to $M(h_0,x_0)$ has been analyzed for each equilibrium point (h_0,x_0). The eigenvalues $\lambda_{1,2}$ were computed as the roots of the following algebraic second order equation

$$det\,(I - \lambda M(h_0,x_0)) = 0 \tag{S14}$$

where I indicated the 2×2 identity matrix and $det()$ indicates the computation of the determinant of a square matrix. When at least one of the eigenvalue had positive real part, the equilibrium point was classified as *unstable*. When both eigenvalues had negative real parts, the equilibrium point was classified as *stable*. Nothing can be however concluded on the stability of the non-linear system, in the cases in which one or both eigenvalues have zero real part (and the other has negative real part). Stable and unstable equilibrium points have been graphically represented as circles and squares in Fig. S1, respectively.

1.2 Single population with non-depressing facilitating synapses

The equations under section "Mean-field Network Description" of the main text can be again simplified under the hypothesis of very fast recovery from depression τ_{rec}. The mean-field firing rate dynamics of a single neuronal population recurrently connected by short term plastic synapses can be rewritten as

$$\begin{cases} \tau\dot{h} = -h + AuE + I_{ext} \\ \dot{u} = (U-u)/\tau_{facil} + U(1-u)E \end{cases} \tag{S15}$$

While this hypothesis is not as realistic as the one of the previous section, it is preparatory for the analysis of the full model. As for the previous case, we consider the derivation of the equilibrium points for h and u as well as the assessment of their stability. Substituting the definitions of equilibrium $\dot{h}=0$ and $\dot{u}=0$ into Eqs. S15, we get

$$\begin{cases} h = AuE + I_{ext} \\ u = U(1+E\tau_{facil})/(1+U\tau_{facil}E) \end{cases} \tag{S16}$$

Using the second equation to replace u as it appears in the first, one obtains an implicit equation in h:

$$h = AU(1+E\tau_{facil})E/(1+U\tau_{facil}E) + I_{ext} \tag{S17}$$

As for Eq. S3, numerical methods can be used for solving Eq. S17 looking for values of h that satisfy the equivalence given a specific set of parameters A, U, τ_{facil}, and I_{ext}. The solution(s) of Eq. S17 can be also graphically interpreted as the intersection(s) in the cartesian plane of two functions of h, $F_1(h)$, and $F_3(h)$ as defined below

$$\begin{cases} F_1(h) = h \\ F_3(h) = AU(1+E\tau_{facil})E/(1+U\tau_{facil}E) + I_{ext} \end{cases} \tag{S18}$$

We observe that by definition of $E=[\alpha(h-\theta)]_+$, $F_3(h)$ is zero when $h \le \theta$. When $h \to +\infty$, the function diverges to infinity, but it can also be approximated by the straight line $F_3(h) \approx \alpha Ah$. We also note that for values of h larger than θ, the first derivative of $F_3(h)$ is positive, indicating that the function is monotonically increasing. In the same range of h, the second derivative is also positive, therefore indicating that the function is concave.

$$\begin{cases} \dot{F_3}(h) = \alpha AU\left[1+2\alpha\tau_{facil}(h-\theta)+\alpha^2\tau_{facil}^2U(h-\theta)^2\right]/[1+\alpha\tau_{facil}U(h-\theta)]^2 \\ \ddot{F_3}(h) = 2\alpha^2\tau_{facil}AU(1-U)/\left[1+\alpha\tau_{facil}U(h-\theta)\right]^3 \end{cases} \tag{S19}$$

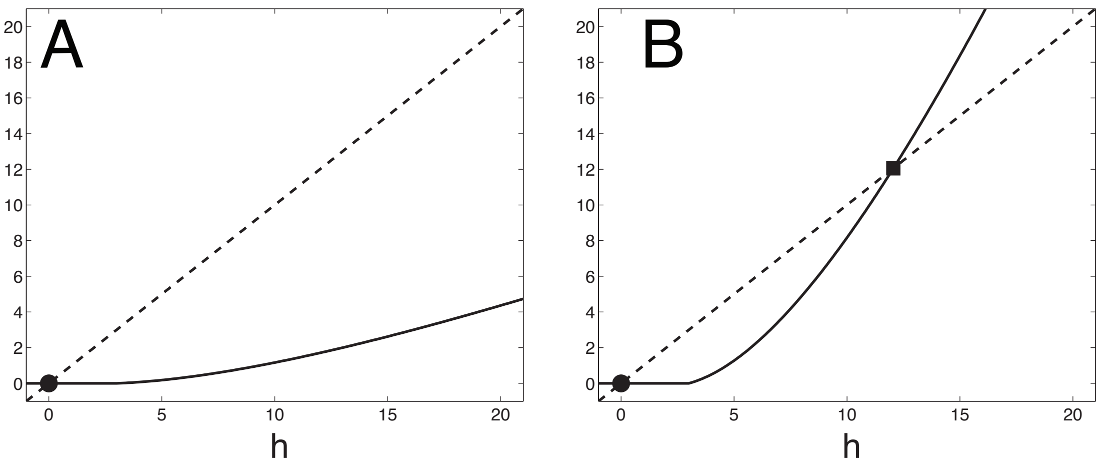

Figure S2. Graphical representation of the function $F_3(h)$ for different values of A. The function $F_3(h)$ has been plotted for different values of the maximal synaptic efficacy A and external input I_{ext}, resulting in one (**A**: $A = 0.5$, $I_{ext} = 0$) or two intersections (**B**: $A = 3.5$, $I_{ext} = 0$). The remaining parameters were: $U = 0.1$, $\tau_{facil} = 500$ msec, $\theta = 3$, $\alpha = 1$. The scripts to generate these plots and to carry out asymptotic analysis on the stability of the equilibrium points, see the text, are available online from the ModelDB (accession number 150211).

As opposed to the previous case, the value of the slope of the tangent line at $F_3(h)$ at $h = \theta$ is not particularly relevant when drawing $F_3(h)$, since at any coordinates $h > \theta$ all the other tangent lines are by definition steeper than it. The minimal slope is $\dot{F}_3(\theta) = \alpha AU$ can then used in combination with the asymptotic approximation $F_3(h) \approx \alpha Ah$ (i.e., the maximal slope is αA). It is then clear that for $0 \leq I_{ext} \leq \theta$ a sufficient and necessary conditions for having always two equilibrium points (i.e., one stable at the value $h = I_{ext}$ when $0 \leq I_{ext} \leq \theta$ and the other unstable) is represented by $\alpha A > 1$ (Figs. S2A,B). All the considerations on how to assess the dynamical stability of these equilibrium points hold and the expression of the *Jacobian* matrix M, whose eigenvalues determine the stability, is given below:

$$M(h_0, u_0) = \begin{pmatrix} (-1+\alpha A u_0)/\tau & (\alpha A U(h_0-\theta))/\tau \\ -\alpha U(1-u_0) & -\tau_{facil}^{-1} - \alpha U(h_0-\theta) \end{pmatrix} \tag{S20}$$

1.3 Single population with short term plastic synapses

In the general case, the mean-field equations of a single neuronal population recurrently connected by short term plastic synapses are given by

$$\begin{cases} \tau\dot{h} = -h + AuxE + I_{ext} \\ \dot{x} = (1-x)/\tau_{rec} - uxE \\ \dot{u} = (U-u)/\tau_{facil} + U(1-u)E \end{cases} \tag{S21}$$

with the neuronal gain function chosen as a threshold-linear relationship between input (mean) current h and output firing rate $E = [\alpha(h-\theta)]_+$. In the main text, we denote the average synaptic efficacy as $J = Aux$. The analysis of this system, including its equilibrium points, has been already given elsewhere [1]. Supporting the necessary condition on the symmetry breaking by long-term plasticities mentioned in the Results section of the main text, here we derive a simple observation on the analytical properties of these equilibrium points. According to the definition, we substitute $\dot{h} = 0$, $\dot{x} = 0$, and $\dot{u} = 0$ into Eqs. S21, and get

$$\begin{cases} h = AuxE + I_{ext} \\ x = 1/(1 + u\tau_{rec}E) \\ u = U(1 + E\tau_{facil})/(1 + U\tau_{facil}E) \end{cases} \tag{S22}$$

By appropriate substitutions of x and of u into the first equation, it is possible to express it as $h = F_4(E(h))$, an implicit equation in h:

$$F_4(E) = \frac{AU(E^{-1} + \tau_{facil})}{E^{-2} + E^{-1}U\tau_{facil} + E^{-1}U\tau_{rec} + U\tau_{facil}\tau_{rec}} + I_{ext}. \tag{S23}$$

We observe that for $h \to +\infty$, $E(h) \to +\infty$ and $F_4(E) \to A\tau_{rec}^{-1} + I_{ext}$, implying the existence of an horizontal asymptote. This intuitively suggests that for any choice of the other parameters compatible with the existence of multiple equilibrium points (i.e., intersections between $F_4(h)$ and the unitary slope line), the uppermost equilibrium point (i.e., always stable) will change its location proportionally to A and to τ_{rec}^{-1}, for the same choice of I_{ext}. Hence, a high value of τ_{rec} as in depressing synapses will give a lower asymptote versus a low value, as in facilitating synapses.

We emphasise that we invoke the separation of time scales for short- and long-term plasticities. As a consequence, in the analysis above we consider A to be fixed and not to change over long time scales as AW. In the main text, we do consider that STDP scales the maximal synaptic efficacy via $W(t)$. Therefore, in accordance to the full mean-field equations (Eqs. 12-15), we can replace A with $AW(t)$ so that the horizontal asymptote can be expressed as a function of t, as $F_4(E) \rightarrow AW(t)\tau_{rec}^{-1} + I_{ext}$. Hence STDP directly affects the stable uppermost equilibrium point.

Let's now consider two independent populations of excitatory neurons, one recurrently connected by short term depressing synapses (i.e., $\tau_{rec} > \tau_{facil}$) and one by short term facilitating synapses (i.e., $\tau_{facil} > \tau_{rec}$) and both receiving identical non-zero external inputs I_{ext}. As for the previous considerations on the horizontal asymptote of $F_4(E)$, for an appropriate choice of A (i.e., large enough to have multiple equilibrium points) or for any value of $I_{ext} > \theta$ the firing rate uppermost equilibrium point of the facilitating population will always be larger than the firing rate uppermost equilibrium point of the depressing population. Together with the specific firing rate dependence of STDP, arising from the triplet-interactions, this consideration rules out that reciprocal motif of short term depressing synapses will outnumber unidirectional motif of facilitating synapses. The stability analysis for the depressing and facilitating populations (with the parameters used in our simulations) is provided in the main text (see Fig. 3D-F).

Assessing the stability of the above system reduces to linearization of the system around the fixed points by the use of a Taylor expansion and the study of the so called *Jacobian* matrix $M(h_0, x_0, u_0)$ of the system, which for the sake of completeness, we report below:

$$\begin{pmatrix} (-1+\alpha A u_0 x_0)/\tau & (\alpha A u_0 (h_0-\theta))/\tau & (\alpha A x_0 (h_0-\theta))/\tau \\ -\alpha u_0 x_0 & -\tau_{rec}^{-1} - \alpha u_0 (h_0-\theta) & -\alpha x_0 (h_0-\theta) \\ \alpha U (1-u_0) & 0 & -\tau_{facil}^{-1} - \alpha U (h_0-\theta) \end{pmatrix} \tag{S24}$$

1.4 The impact of recurrent inhibition

We extend the description of the system given in Eqs. S1 to the case where recurrent inhibition is explicitly accounted for. The mean-field firing rate dynamics of two neuronal populations, one excitatory and one inhibitory, recurrently connected by short term excitatory plastic synapses and by frequency-independent inhibitory synapses (as in Fig. S3), can be rewritten as

$$\begin{cases} \tau_e \dot{h}_e = -h_e + A_{ee} U x E - A_{ei} I + I_{ext} \\ \tau_i \dot{h}_i = -h_i + A_{ie} U x E \\ \dot{x} = (1-x)/\tau_{rec} + U x E \end{cases} \tag{S25}$$

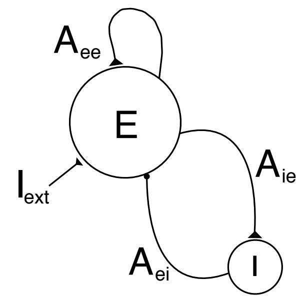

Figure S3. mean-field description for two recurrently connected populations of excitatory and inhibitory neurons. Excitatory neurons are recurrently connected by short term plastic synapses and project to a population of inhibitory neurons. Inhibitory neurons project back to the excitatory population with non-plastic, linear synapses.

with $E = [\alpha_e(h_e - \theta_e)]_+$ and $I = [\alpha_i(h_i - \theta_i)]_+$. We consider here the derivation of the equilibrium points for h_e, h_i, and x and we indicate how their stability was assessed. Substituting the conditions $\dot{h}_e = 0$, $\dot{h}_i = 0$, and $\dot{x} = 0$ in Eqs. S25, we obtain three non-linear algebraic equations

$$\begin{cases} h_e = A_{ee}UxE - A_{ei}I + I_{ext} \\ h_i = A_{ie}UxE \\ x = 1/\left(1 + U\tau_{rec}E\right) \end{cases} \tag{S26}$$

Using the second and third equations to replace x and I in the first we get an implicit equation in the unknown h_e:

$$h_e = \frac{A_{ee}UE}{1 + U\tau_{rec}E} - A_{ei}\left[\alpha_i\left(\frac{A_{ie}UE}{1 + U\tau_{rec}E} - \theta_i\right)\right]_+ + I_{ext} \tag{S27}$$

We make the assumption that the synaptic coupling from the excitatory to the inhibitory population is sufficiently strong $A_{ie} > \tau_{rec}\theta_i$ so that short term depression of that pathway does not prevent steady recruitment of inhibition at higher firing rates of the excitatory population. We also assume for simplicity that recurrent excitation is also sufficiently strong so that $A_{ee} > \alpha_i A_{ei} A_{ie}$. Under these hypotheses, when E is below a certain value, i.e., $E < \theta_i/\left[U\left(A_{ie} - \tau_{rec}\theta_i\right)\right]$, the above implicit equation coincides with Eq. S3 and it can be written as if inhibition was not present:

$$h_e = A_{ee}UE/\left(1 + U\tau_{rec}E\right) + I_{ext} \tag{S28}$$

Instead, above that critical value for E (i.e., and therefore for h_e) Eq. S27 does not change formally apart from its coefficients:

$$h_e = \hat{A}_{ee} U E / \left(1 + U \tau_{rec} E\right) + \hat{I}_{ext} \tag{S29}$$

with $\hat{A}_{ee} = A_{ee} - \alpha_i A_{ei} A_{ie}$ and $\hat{I}_{ext} = I_{ext} + \alpha_i \theta_i A_{ei}$. It is easy to verify that $0 < \hat{A}_{ee} < A_{ee}$ and $\hat{I}_{ext} > I_{ext}$.

The critical value for the recurrent inhibitory inputs to affect the excitatory population can be translated into a condition on h_e, i.e., $h_e > \theta_e + \theta_i / \left[\alpha_e U \left(A_{ie} - \tau_{rec}\theta_i\right)\right]$. Under our previous hypothesis, such a critical value for h_e is always larger than the threshold θ_e for the activation of the excitatory neurons. There will exist a range of activation for h_e above θ_e, where the impact of inhibition is negligible. For larger activation h_e, inhibition kicks by step-wise decreasing the parameter $\hat{A}_{ee}$.

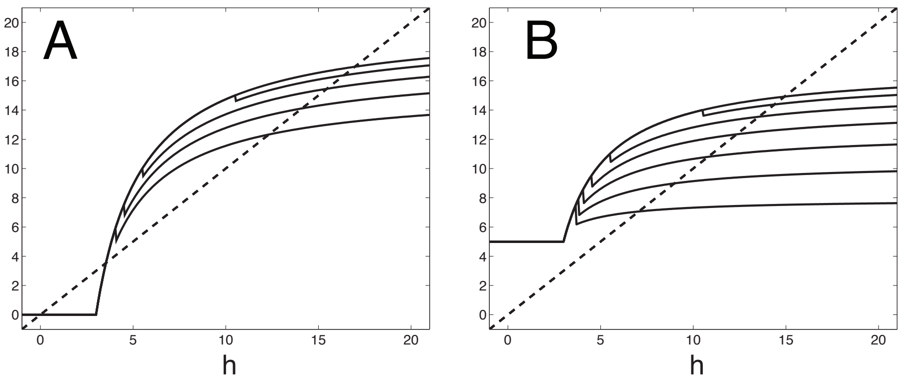

Figure S4. Graphical representation of the equivalent of functions $F_2(h)$ and $F_3(h)$ in the presence of inhibition. Increasing the coupling between the excitatory and the inhibitory population substantially bends down the curves previously analyzed as $F_2(h)$ and $F_4(h)$, lowering the firing rate of the equilibrium points ($A_{ei} = A_{ie}/10$; **A**: $I_{ext} = 0$, **B**: $I_{ext} = 5$).

All in all, the presence of recurrent inhibition in the system does not alter qualitatively the conclusions on the existence of equilibrium points of the mean-field description. The statement on the separation of the uppermost equilibrium points, associated respectively to the short term depressing and the short term facilitating networks, remains true since

the horizontal asymptote shares the same indirect proportionality relationship with the time constant τ_{rec} of recovery from depression.

1.5 Single population with mixed synapses

We consider the special case of a homogeneous network of neurons, whose connections to distinct target postsynaptic neurons can be simultaneously short term depressing and short term facilitating. For the sake of simplicity and for distinguishing this case from the mixed populations studied in the main text (see Fig. 4), neurons are assumed to be indistinguishable from each other. However, every neuron has a certain probability p_D to establish a short term depressing connection with its postsynaptic target. The same neuron has probability $1 - p_D$ to establish a short term facilitating connection to another postsynaptic neurons. Under these simplifying hypotheses, and by definition of conditional expected value [5], the mean-field firing rate dynamics of the neuronal population recurrently connected by short term plastic synapses is

$$\begin{cases} \tau \dot{h} = -h + A\left[p_D u_D x_D + (1-p_D) u_F x_F\right] E + I_{ext} \\ \dot{x_D} = \left(1 - x_D\right) / \tau_{rec_D} - u_D x_D E \\ \dot{u_D} = \left(U_D - u_D\right) / \tau_{facil_D} + U_D \left(1 - u_D\right) E \\ \dot{x_F} = \left(1 - x_F\right) / \tau_{rec_F} - u_F x_F E \\ \dot{u_F} = \left(U_F - u_F\right) / \tau_{facil_F} + U_F \left(1 - u_F\right) E \end{cases} \tag{S30}$$

The cases $p_D = 0$ and $p_D = 1$ have been already examined in the previous sections. For intermediate values of p_D, an evaluation of the equilibria of Eqs. S30 has been carried out numerically, resulting in a qualitatively similar behavior to the extreme cases, with the location of the (stable) equilibrium points to be intermediate between those of a short term depressing neuronal network and those of a short term facilitation neuronal network. As expected, for increasing values of p_D the location of all equilibrium points of $E(h)$ (if any) decreases monotonically.

1.6 Emergence of motifs for identical initial couplings

In the main text, we indicate that a specific initial configuration for the intra- and across-population synaptic efficacies is required for the emergence of connectivity motif in heterogenous networks. In particular, we require that connections across populations should be weaker than connections within populations. In this section we partially relax this condition by showing that an appropriate external stimulation protocol can shape an network where maximal synaptic efficacies are initially equal (i.e., $W_{FF} = W_{DD} = W_{FD} = W_{DF}$) to the required specific configuration for the emergence of the motif. We remind

the reader that for simplicity have dropped the notation ˆ from mean-field variables, and that without loss of generality we set $A = 1$, see section "Mean-field simulation of a heterogeneous network with short- and long-term plasticity" of the main text.

We assume that the two subpopulations receive a common external input and an alternating pulsed stimulus component. As in a recurring traveling wave of external activity, each subpopulation is alternatively exposed to an pulsating input component, so that both facilitatory and depressing subpopulations are activated but never at same time.

Due to intrinsic subpopulation properties, determined by short term synaptic plasticities and reviewed in the previous sections, and as a direct consequence of the associative character of long-term plasticity discussed in the main text, this stimulation protocol leads to stronger synaptic coupling within the populations and weaker synaptic coupling across-populations (see Figsfig:weightForm). As discussed in the results of the main text, such a configuration is retained indefinitely even in the absence of external alternating stimulation.

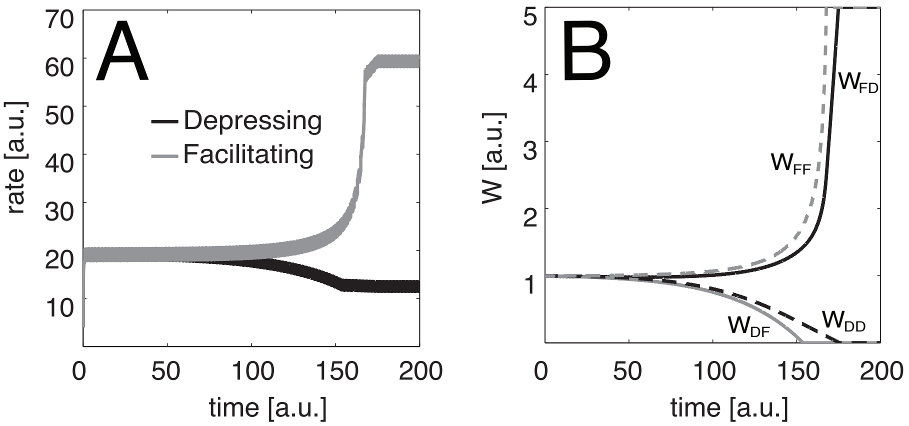

Figure S5. Formation of asymmetric synapses among and across populations. The subpopulations D and F receive a common input current ($I_C = 12.5$) and an alternating stimulation component (I_{ED}, I_{EF}) oscillating periodically between two amplitude levels (i.e., 2 and 0) every 1 [a.u.] of time: whenever $I_{ED} = 0$, $I_{EF} = 2$ and vice versa. The temporal evolution of the firing rate of each population is shown in **A**, while the corresponding long-lasting plastic changes of the maximum synaptic coupling (i.e., W_{FF}, W_{DD}, W_{FD}, W_{DF}) is plotted in **B** upon initialization to the same value (i.e., 1). The same final configuration is generally obtained even by randomly initializing W_{FF}, W_{DD}, W_{FD}, and W_{DF} by a Gaussian distribution with mean 1 and standard deviation 0.001, for 90 out of 100 simulation runs, demonstrating a degree of robustness.

To understand why such a stimulation protocol succeeds in developing the desirable coupling asymmetries we must examine the maximal synaptic efficacy in its mean-field formulation (see main text). Let's assume that, due to the external stimulation, the firing rate E_D of the depressing subpopulation is larger than the firing rate E_F of the facilitating subpopulation (i.e., say, $E_D = kE_F$, with $k > 1$). This configuration of firing rates is forced by the external input component, which alternates in time and across the subpopulations. Under these conditions, the STDP would modify intra-population synaptic coupling J_{DD} as

$$\Delta W_{DD} = -A_2^- \tau_{o_1} E_D^2 + A_3^+ \tau_{r_1} \tau_{o_2} E_D^3 \tag{S31}$$

while for the synaptic couplings across-population, the STDP results into

$$\Delta W_{DF} = -\frac{1}{k} A_2^- \tau_{o_1} E_D^2 + \frac{1}{k} A_3^+ \tau_{r_1} \tau_{o_2} E_D^3 \tag{S32}$$

$$\Delta W_{FD} = -\frac{1}{k} A_2^- \tau_{o_1} E_D^2 + \frac{1}{k^2} A_3^+ \tau_{r_1} \tau_{o_2} E_D^3 \tag{S33}$$

It is now easy to prove that $\Delta W_{DD} > \Delta J_{DF} > \Delta W_{FD}$, hence identical initial values for W_{DD}, W_{DF}, and W_{FD} would lead to $W_{DD} > W_{DF}$ and to $W_{DD} > W_{FD}$. Similar considerations can be repeated when the firing rate E_F of the facilitating subpopulation is larger than the firing rate E_D of the depressing subpopulation (i.e., say, $E_F = kE_D$, with $k > 1$), concluding that the stimulation protocol would shape synaptic efficacies as $W_{DD} > W_{DF}$, $W_{DD} > W_{FD}$, $W_{FF} > W_{DF}$, and $W_{FF} > W_{FD}$, therefore leading to stronger intra-population coupling in comparison to across-population coupling, as shown in Fig. S5B.

1.7 Pair-based alternative STDP model

By appropriately choosing A_2^+, A_2^-, and setting to zero both A_3^+ and A_3^-, earlier phenomenological models of pair-based STDP can be rephrased as a special case of the triplet-based model. For the pair-based STDP each neuron of the network needs only two indicator variables, i.e., q_1 and o_1, instead of four. In the lack of any firing activity of the jth neuron, those variables exponentially relax to zero:

$$\tau_{q_1} \dot{q}_{1_j} = -q_{1_j} \qquad \tau_{o_1} \dot{o}_{1_j} = -o_{1_j} \tag{S34}$$

As the jth neuron fires the variables must be instantaneously updated. For such update rule there are two distinct scenarios determining how successive pre-post or post-pre events interact and affect synaptic efficacy: i) *all-to-all* spike pairs interactions,

$$q_{1_j} \to q_{1_j} + 1 \qquad o_{1_j} \to o_{1_j} + 1 \tag{S35}$$

and ii) *nearest-spike* interactions,

$$q_{1_j} \rightarrow 1 \qquad o_{1_j} \rightarrow 1. \tag{S36}$$

where the update rules do not allow accumulation of effects. Finally, when the jth neuron spikes, the following updates are performed over all the indexes i:

$$\begin{cases} W_{ij} \rightarrow W_{ij} - \eta A_2^- o_{1_i}(t) \\ W_{ji} \rightarrow W_{ji} + \eta A_2^+ q_{1_i}(t) \end{cases} \tag{S37}$$

Following Pfister and Gerstner [6], pair-based models of STDP with all-to-all interactions must be excluded, as they do not reproduce realistic (i.e., BCM) features of synaptic plasticity. We then consider the triplet-based STDP model and alter some of its parameters as it follows: $A_3^+ = A_3^- = 0$, $A_2^+ = 4.510^{-3}, A_2^- = 7.110^{-3}$. We also replaced the *all-to-all* spike pairs interactions by a *nearest spike* interaction, by modifying the update rule for q_1 and r_1 (and for q_2 and r_2, although those state variables are anyway irrelevant, upon setting $A_3^+ = A_3^- = 0$). By doing so we obtain the pair-based STDP plasticity rule matching the exact same temporal window of the STDP triplet model employed here. In order to prove that there is correspondence in terms of the temporal window, but altered frequency-dependence, we subjected both the triplet-based and the pair-based models to 75 pairing events at low frequency 10 Hz. The STDP temporal windows at such a frequency are undistinguishable from each other (see main text). The frequency-dependence is computed across the same number of pairing events, imposing a pre-post or post-pre delay of 10 msec.

1.8 Triplet-based anti-STDP model

This model is obtained from the triplet-based by setting $A_3^+ = 7.110^{-3}$, $A_3^- = 6.110^{-3}$, $A_2^+ = 0$, and $A_2^- = 3.510^{-3}$, leaving unchanged the update rules for q_1, q_2, o_1, and o_2 as in the original triplet model, and by modifying the actual weight update equations as it follows: when the jth neuron spikes, the following updates are performed for all the indexes i:

$$\begin{cases} W_{ij} \rightarrow W_{ij} + \eta o_{1_i}(t) \left[A_2^+ + A_3^+ q_{2_j}(t-\epsilon)\right] \\ W_{ji} \rightarrow W_{ji} - \eta q_{1_i}(t) \left[A_2^- + A_3^- o_{2_j}(t-\epsilon)\right] \end{cases} \tag{S38}$$

Note that this is only a tentative proposal for an anti-STDP rule since experimental of data are not yet available for all induction protocols earlier employed for STDP. In particular, we ignore the frequency-dependency of the anti-STDP and by the new parameter set we roughly leave it untouched.

2 Time-coding inputs and unidirectional motifs

This section examines the case in which by external activity, in the form of a temporal coded inputs, the emergence of unidirectional motifs occur. This analysis is based on the analysis found in [7] with minor adaptations for our specific choice of synaptic model. The triplet-based STDP model for $A_2^+ = A_3^- = 0$ is given by the following equation (see also Methods in main text). As the jth neuron fires, over all indexes i

$$\begin{cases} \dot{W}_{ij} = -\eta A_2^- o_{1_i}(t) \\ \dot{W}_{ji} = \eta A_3^+ q_{1_i}(t) o_{2_j}(t-\epsilon) \end{cases} \tag{S39}$$

According to the main text protocol, we assume that N neurons form conceptually a ring and an external stimulus is forcing one after the other to fire with an interval of τ. We then evaluate the term $o_{1_i}(t)$, which is a low pass filter signal of the activity of neuron i at the time of the pre-synaptic spike, represented by a δ function X_j:

$$\int_{cycle} o_{1_i}(t) X_j dt = \sum_{n=0}^{\infty} \exp\left(-\frac{[(j-i)\mathrm{mod}N]\tau + nN\tau}{\tau_{o_1}}\right), \tag{S40}$$

where the term $[(j-i)\mathrm{mod}N]\tau$ (for $n=0$) is the time that has passed from when neuron i fired in this last cycle, the term $[(j-i)\mathrm{mod}N] + N\tau$ (for $n=1$) is the time that has passed from the previous cycle etc. We note that the term $\sum_{n=0}^{\infty}\exp(-\frac{nN\tau}{\tau_{o_1}})$ is a geometrical series. Then, after a long time, the synapse W_{ij} has been decreased by the term

$$\Delta W_{ij}^{\infty} = -\eta A_2^- \exp\left(-\frac{[(j-i)\mathrm{mod}N]\tau}{\tau_{o_1}}\right) \frac{1}{1-\exp\left(-\frac{N\tau}{\tau_{o_1}}\right)} \tag{S41}$$

This tells us that the firing of the neuron j will depress all synaptic efficacies from j to all other neurons, but most strongly the connection to the neuron that fired just before, i.e., with index $j-1$ and less strongly the connection to the neuron that will fire just after j, i.e., the neuron with index $j+1$.

Similarly, we evaluate the terms $q_{1_i}(t)o_{2_j}(t-\epsilon)$ when the j neuron fires:

$$\int_{cycle} q_{1_i}(t) o_{2_j}(t-\epsilon) X_j dt == \sum_{n=0}^{\infty} \exp\left(\frac{[(j-i)\mathrm{mod}N]\tau + nN\tau}{\tau_{q_1}}\right) \sum_{n=0}^{\infty} \exp - \frac{N\tau(n+1)}{\tau_{o_2}} \tag{S42}$$

resulting in

$$\Delta W_{ji}^{\infty} = \eta A_3^+ \exp\left(-\frac{[(j-i)\mathrm{mod}N]\tau}{\tau_{q_1}}\right) \frac{1}{1-\exp\left(-\frac{N\tau}{\tau_{q_1}}\right)} \frac{1}{\exp\left(\frac{N\tau}{\tau_{o_2}}\right)-1} \tag{S43}$$

which tells us that the firing of the neuron j will potentiate synaptic efficacies from all other neurons to j, but most strongly the connection that it receives from the neuron that fired just before, i.e with index $j-1$ and less strongly the connection tag it receives from the neuron that will fire just after j, i.e., the neuron with index $j+1$.

These asymmetries in the potentiation and depression of the synaptic pairs lead to the conclusion that while $W_{j(j-1)}$ will be potentiated at the end of the cycles $W_{(j-1)j}$ will be depressed, leading to unidirectional connectivity between neighboring neurons.

3 Statistics of the "symmetry index"

In order to quantify and describe concisely the symmetries of the emerging network connectivity matrix $[A_{ij}]$ of size $N \times N$, we defined the following quantity (but see, e.g., , [8] for alternative definitions):

$$s = 1 - \frac{1}{(0.5N\,(N-1) - M)} \sum_{i=1}^{N} \sum_{j=i+1}^{N} |A^*_{ij} - A^*_{ji}| \qquad \text{(S44)}$$

The index s intuitively represents the mean absolute difference between elements that are on symmetric positions, with respect to the diagonal of the matrix. By definition, the elements A^*_{ij} are obtained from A_{ij} upon first normalizing its numerical values to the maximal allowed A_{max} and then clipping them to a lower fraction h. For instance, choosing $h = 2/3$, if $A_{ij} > 2/3A_{max}$ then $A^*_{ij} = A_{ij}/A_{max}$ and otherwise $A^*_{ij} = 0$. In the Eq. S44, M represents the number of null pairs $\{A^*_{ij}, A^*_{ji}\} = \{0, 0\}$ as a consequence of clipping. Then, s can be rewritten in terms of an arithmetic average of a set of K observations of a random variable q:

$$s = 1 - \frac{1}{K} \sum_{i=1}^{K} q_i \qquad \text{(S45)}$$

Assuming for simplicity that each element of $[A_{ij}]$ is independently drawn from a uniform distribution (i.e., between 0 and A_{max}), the probability density distribution of q, $f_q(Q)$, can be derived analytically [5]. Because the arithmetic average is an unbiased estimator of the expected value of the random variable it samples (i.e., in this case q) [5], most of the statistical properties of s can be immediately derived from the distribution of q. First of all, the expected value of s is given by the expression below

$$\langle s \rangle = A_{max} \left(1 - \frac{1-h}{1-h^2} \left[\frac{(1-h)^2}{3} + h(1+h) \right] \right) \qquad \text{(S46)}$$

Deriving the expression for the variance is less straightforward and requires estimating the expected value of $1/K$. In fact K coincides with the number of terms in the double

sum of Eq. S44 and it is by definition not a fixed quantity but a realization of a binomial random variable. An approximated expression for the variance of s is then

$$Var\{s\} \approx A_{max}^2 \frac{2}{N(N-1)(1-h^2)} \left(1 + \frac{2h^2}{N(N-1)(1-h^2)}\right) Var\{q\} \tag{S47}$$

where

$$Var\{q\} = \frac{1}{1-h^2} \left(\frac{(1-h)^4}{6} + \frac{2h(1-h^3)}{3}\right) - (1 - \langle s \rangle)^2 \tag{S48}$$

The validity of all the above expressions have been tested and validated numerically, directly estimating the average and variance of s across thousands of uniform random matrices $[A_{ij}]$ for several values of h in the range $[0.1; 0.9]$ and finding an excellent agreement.

Finally, from the Central Limit Theorem [5], we can expect the density distribution of s to be approximately Gaussian at least for small values of h. By the above statistical expressions, when studying the impact of short- and long-term synaptic plasticity in shaping microcircuit connectivity motif, we expressed the significance of the observed values of s as the chance level, i.e., the (Gauss-distributed) probability that the observed value of s could be obtained by chance from a random uniform matrix.

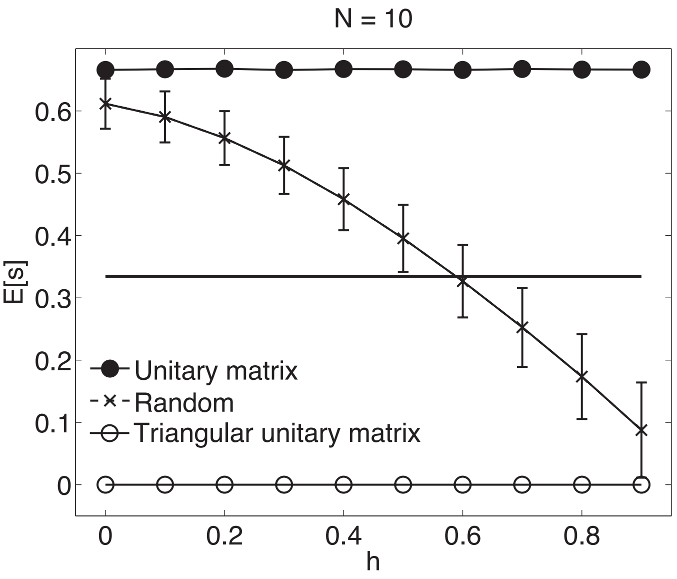

Figure S6. Impact of the clipping threshold parameter h on the symmetry measure s. The continuous trace and $\times$ markers show the expectation of the symmetry index for a random connectivity matrix with 20% of its elements randomly pruned over 10000 samples of 10×10 matrices, with error bars indicating the standard deviation. The simulations were repeated for a unitary matrix (i.e., leading to the maximum possible value for s) and for a upper triangular unitary matrix (i.e., leading to the minimum possible value for s). The value of $h = 2/3$ used in this work, chosen for consistence to earlier works, leads to a middle point between the two extremes considered and thus provide a good discriminating condition when using the statistics of a random matrix as a null hypothesis.

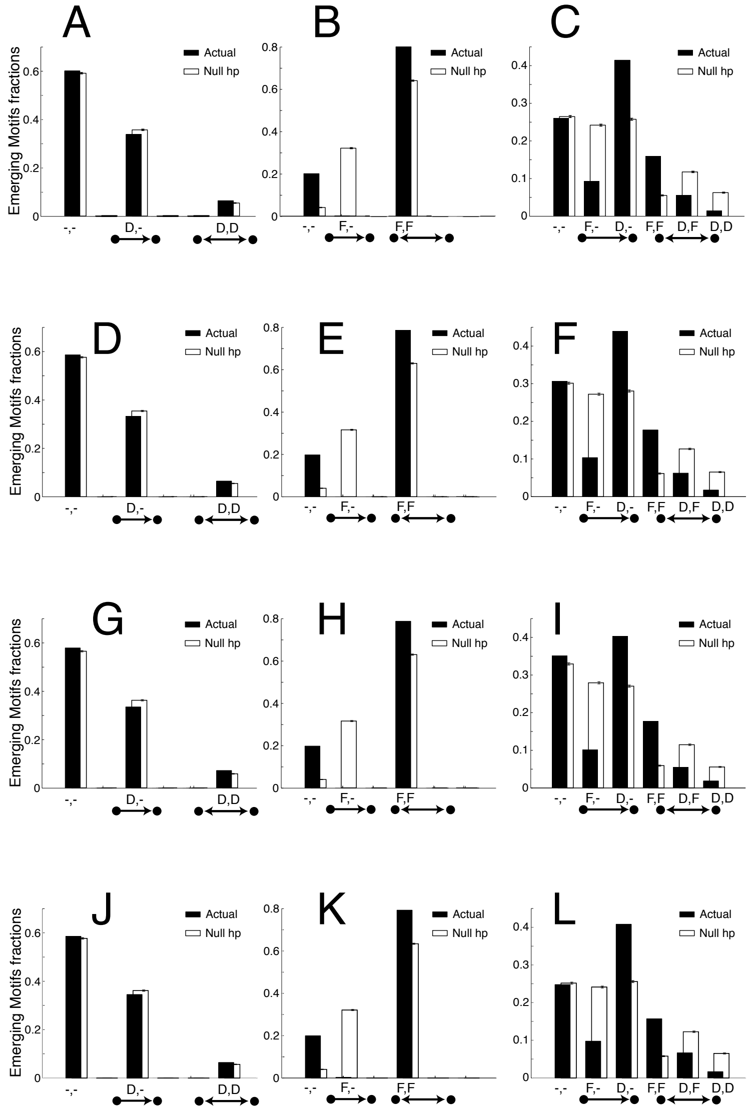

Figure S7. External stimulation protocols and models details do not affect motif emergence. The figure examines the fraction of motif emerging when neurons receive periodic input wave-like stimulation (**A,B,C**). When the periodic stimulation is omitted (**D,E,F**), as well as when each neuron receives instead a ten percent shared random background inputs with each other (**G,H,I**), very similar results emerge. The simulations of panels **D,E,F**, when repeated without spike-frequency adaptation mechanisms from each unit of the networks (**J,K,L**), still give rise to the same results.

References

1. Barak O, Tsodyks M (2007) Persistent activity in neural networks with dynamic synapses. PLoS Comput Biol 3: e35.

2. Tsodyks M (2005) Methods and models in neurophysics, Elsevier, chapter Synaptic Dynamics. pp. 245-66.

3. Strogatz S (1994) Nonlinear dynamics and chaos. Reading, MA (USA): Addison Wesley.

4. Press W, Teukolsky S, Vetterling W, Flannery B (2007) Numerical Recipes: The Art of Scientific Computing. Cambridge University Press.

5. Papoulis A, Pillail SU (2002) Probability, random variables, and stochastic processes. McGraw-Hill, 4th edition.

6. Pfister JP, Gerstner W (2006) Beyond pair-based STDP: A phenomenological rule for spike triplet and frequency effects. MIT Press, volume 17 of *Advances in Neural Inf. Proc. Syst.*, pp. 1409-16.

7. Clopath C, Buesing L, Vasilaki E, Gerstner W (2010) Connectivity reflects coding: A model of voltage-based stdp with homeostasis. Nat Neurosci 13: 344-52.

8. Huber L, Baker F (1979) Evaluating the symmetry of a proximity matrix. Quality and Quantity 13: 77-84.
